# ChimPipe: accurate detection of fusion genes and transcription-induced chimeras from RNA-seq data

**DOI:** 10.1186/s12864-016-3404-9

**Published:** 2017-01-03

**Authors:** Bernardo Rodríguez-Martín, Emilio Palumbo, Santiago Marco-Sola, Thasso Griebel, Paolo Ribeca, Graciela Alonso, Alberto Rastrojo, Begoña Aguado, Roderic Guigó, Sarah Djebali

**Affiliations:** 1 Centre for Genomic Regulation (CRG), The Barcelona Institute of Science and Technology, Dr. Aiguader 88, Barcelona, 08003 Spain; 2 Universitat Pompeu Fabra (UPF), Barcelona, Spain; 3 Joint IRB-BSC Program in Computational Biology, Barcelona Supercomputing Center (BSC), Jordi Girona 31, Barcelona, 08034 Spain; 4 Centro Nacional de Análisis Genómico, Baldiri Reixac, 4, Barcelona Science Park - Tower I, Barcelona, 08028 Spain; 5 Integrative Biology, The Pirbright Institute, London, GU24 0NF UK; 6 Centro de Biología Molecular Severo Ochoa (CSIC - UAM), Nicolás Cabrera 1, Cantoblanco, Madrid, 28049 Spain; 7 Institut Hospital del Mar d’Investigacions Mediques (IMIM), Barcelona, 08003 Spain; 8 GenPhySE, Université de Toulouse, INRA, INPT, ENVT, Castanet Tolosan, France

**Keywords:** Chimera, Transcript, Fusion gene, RNA-seq, Benchmark, Cancer, Simulation, Isoform, Splice junction

## Abstract

**Background:**

Chimeric transcripts are commonly defined as transcripts linking two or more different genes in the genome, and can be explained by various biological mechanisms such as genomic rearrangement, read-through or trans-splicing, but also by technical or biological artefacts. Several studies have shown their importance in cancer, cell pluripotency and motility. Many programs have recently been developed to identify chimeras from Illumina RNA-seq data (mostly fusion genes in cancer). However outputs of different programs on the same dataset can be widely inconsistent, and tend to include many false positives. Other issues relate to simulated datasets restricted to fusion genes, real datasets with limited numbers of validated cases, result inconsistencies between simulated and real datasets, and gene rather than junction level assessment.

**Results:**

Here we present ChimPipe, a modular and easy-to-use method to reliably identify fusion genes and transcription-induced chimeras from paired-end Illumina RNA-seq data. We have also produced realistic simulated datasets for three different read lengths, and enhanced two gold-standard cancer datasets by associating exact junction points to validated gene fusions. Benchmarking ChimPipe together with four other state-of-the-art tools on this data showed ChimPipe to be the top program at identifying exact junction coordinates for both kinds of datasets, and the one showing the best trade-off between sensitivity and precision. Applied to 106 ENCODE human RNA-seq datasets, ChimPipe identified 137 high confidence chimeras connecting the protein coding sequence of their parent genes. In subsequent experiments, three out of four predicted chimeras, two of which recurrently expressed in a large majority of the samples, could be validated. Cloning and sequencing of the three cases revealed several new chimeric transcript structures, 3 of which with the potential to encode a chimeric protein for which we hypothesized a new role. Applying ChimPipe to human and mouse ENCODE RNA-seq data led to the identification of 131 recurrent chimeras common to both species, and therefore potentially conserved.

**Conclusions:**

ChimPipe combines discordant paired-end reads and split-reads to detect any kind of chimeras, including those originating from polymerase read-through, and shows an excellent trade-off between sensitivity and precision. The chimeras found by ChimPipe can be validated in-vitro with high accuracy.

**Electronic supplementary material:**

The online version of this article (doi:10.1186/s12864-016-3404-9) contains supplementary material, which is available to authorized users.

## Background

Chimeric transcripts or chimeras are transcripts whose sequence originates from two or more different genes in the genome [1], and can be explained by several different biological mechanisms at the genomic or the transcriptional level. For its historical relation to cancer, the most well known mechanism is genomic rearrangement. This process brings two genes that are far apart in the germline genome close to each other, and in the same direction, in the cancer genome. The fusion gene thus created can have a deleterious role, either as a protein or as a transcript [1, 2]. Aside from chimeras that are important for their known role in cancer, there are other functional *transcriptional* mechanisms that can also explain the formation of chimeras in normal or tumour cells: polymerase read-through and trans-splicing [1].

As indicated by its name, polymerase read-through occurs when the polymerase reads through one gene into the next, therefore creating a chimera between two adjacent genes. Initially thought to be an exception, this mechanism was found to be widespread in mammals when large collections of ESTs (Expressed Sequence Tags) and cDNAs (complementary DNA) became available and were mapped to the genome [3–5], and when the ENCODE (ENCyclopedia Of DNA Elements) consortium systematically surveyed the transcriptome associated to annotated protein coding genes [6–9]. Read-throughs occur between annotated exons of adjacent genes, preferentially between the penultimate exon of the upstream (5’) gene and the second exon of the downstream (3’) gene [3], resulting in new proteins containing domains from the two parent genes, therefore increasing the diversity of a species proteome [1, 3, 4, 10, 11]. They are also largely conserved across vertebrates [11, 12], and could be a way to regulate the expression of one or both parent genes [12].

Trans-splicing is a splicing mechanism that, unlike the well known cis-splicing, occurs between two different pre-messenger RNA (pre-mRNA) molecules close in the three dimensional (3D) space of the nucleus and thought to belong to the same ‘transcription factory’. If the two pre-mRNAs come from two different genes, a transcriptional chimera is generated [1, 13–16]. The two connected genes can therefore be located distally from each other in the genome, however the chimeric junction must have canonical splice sites. Initially thought to be restricted to trypanosomatidae, this mechanism has gained interest in human research since several studies have found chimeras between genes on different chromosomes or strands, without evidence of underlying genomic rearrangements [13, 14, 16]. One hypothesis is that such trans-spliced transcripts occurring in normal cells would trigger a genomic rearrangement, which will in turn produce a higher quantity of these transcripts (although through a different mechanism), eventually leading to tumorigenesis [13].

But chimeras can also be non-functional, either because they are biological noise from the transcriptional machinery, or because they are technical artefacts from Reverse Transcriptase polymerase chain reaction (RT-PCR) based assays. A biological source of artefactual chimeras is polymerase transcriptional slippage through short homologous sequences (SHS), where the polymerase switches template (or pre-mRNA), in the presence of a short sequence with high similarity to the one it is currently transcribing, in another gene close in the 3D space [17]. This mechanism is reminiscent of the reverse transcriptase (RT) template switching, which can also produce artefactual chimeras in RT-PCR- based experiments [18, 19]. Note that in both cases the chimeric junctions will harbor SHS and non canonical splice sites, however those are not sufficient conditions for a chimera to be an artefact, since RNAse protection assay experiments, which are not RT-PCR-based, have confirmed a number of them [9].

The importance of chimeras lies in their ability to create novel transcripts and proteins, therefore potentially altering the phenotype of cells, individuals or groups of individuals [1, 3, 4, 10, 20]. In the field of cancer, some fusion genes are cancer driver events and can be used as biomarkers or even lead to effective treatment - for instance BCR-ABL1 in chronic myeloid leukemia (CML) [21] or TMPRSS2-ERG in prostate cancer [22, 23]. However not all cancer related chimeras result from genomic rearrangements, since some of them can originate from read-through [24–28], and this mechanism could also be the most prevalent one for certain cancer types, such as CLL [29]. Although chimeras’ function have mostly been investigated in relation to cancer, chimeras can also be functionally important in other fields. For instance a chimera produced by trans-splicing, TsRMST, has been shown to interact with pluripotency related transcription factors to control cells’ pluripotency [15], and the knock-down of two widely expressed chimeras, CTBS-GNG5 and CTNNBIP1-CLSTN1, in non-neoplastic cell lines, resulted in significant reduction in cell growth and motility [30].

These events were previously detected by RT-PCR-based methods such as EST alignment to the genome [5, 12], or RACEarray followed by RT-PCR, cloning and sequencing [7, 9], however RNA-seq has been shown to be both a more precise and a more sensitive detection method [24]. A growing number of bioinformatic methods have been created to detect chimeras amongst such datasets [31–39].

**Fig. 1:**
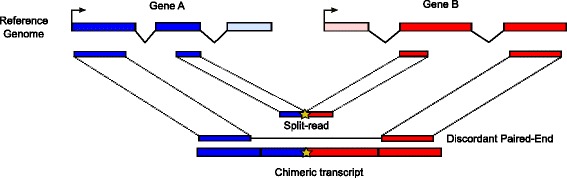
Two types of RNA-seq reads for chimera detection. This picture shows a chimeric transcript (*bottom*) made from exons of two genes, A and B, depicted in *blue* and *red* respectively (*top*). This chimeric transcript is supported by two types of reads: a split-read and a discordant paired-end read, that we depict aligned both on the genome (*middle-top*) and on the transcriptome (*middle-bottom*). The chimeric junction position on the transcriptome is highlighted by a *yellow star* both in the split-read and in the chimeric transcript

These state-of-the art programs usually include 3 steps: (1) mapping and filtering for chimeric reads, (2) chimeric junction detection, and (3) chimera assembly and filtering [40]. They rely heavily on an underlying mapper to map the reads to the genome (and optionally to the transcriptome), and make use of two kinds of reads for chimera detection: (1) discordant paired-end (PE) reads, i.e. paired-end reads where the two mates map in a way that is not consistent with annotated gene structure, e.g. on different chromosomes, and (2) ‘split’ reads, i.e. reads that do not map contiguously to the genome but have to be split or fragmented into several blocks (usually two) to map to the genome (Fig. 1). In addition, the use of one or two kinds of reads for chimeric junction detection allows one to define 3 classes of approaches: (1) the whole paired-end approach, (2) the direct fragmentation approach, and (3) the paired-end + fragmentation approach [41].

Benchmarking of these programs has shown a high false positive rate and a poor intersection between their outputs on the same dataset [42, 43]. On the other hand these programs are usually developed to detect fusion genes in human cancer, and are therefore not always able to detect read-through events and to work on species other than human. In addition, these programs are not always able to predict multiple isoforms per gene pair, and more importantly to provide base pair resolution, preventing their downstream functional validation. To address these problems we present ChimPipe, a modular method which uses the paired-end + fragmentation approach and a set of stringent filters, to reliably detect both transcriptional chimeras and fusion genes from Illumina paired-end RNA-seq data from both normal and tumor samples, in any eukaryotic species with a genome and an annotation available. The advantage of the paired-end + fragmentation approach is the complementarity of the two types of reads used, with the first ones relatively easy to find but only providing a rough indication of the connected regions, and the second ones more error prone but providing the exact chimeric junction coordinates. The biggest difference between ChimPipe and other tools of the field is its independent generation of split-reads and discordant paired-end reads. Programs using the paired-end + fragmentation approach usually first find discordant paired-end reads, then make an exon-exon junction database from them, and finally map the yet unmapped reads to this database. They are therefore not able to find split-reads that do not have associated discordant PE reads. Contrary to these programs, ChimPipe finds split-reads and discordant paired-end reads independently, defines chimeric junctions based on the first ones (known to be more sensitive) and uses the second ones as a way to reduce the false positive rate (although their use is not compulsory). The second biggest difference with other tools is the fact that ChimPipe uses mapping tools (GEMtools RNA-seq pipeline and GEM RNA mapper) that guarantee an exhaustive mapping search given the input parameters, which again allows for a higher initial sensitivity. Our combination of filters may also be more complete than for other tools since it is based on chimera expression, gene annotation, mitochondrial read removal, and homology between connected genes. In practice it allows ChimPipe’s false positive rate to be rather low, but not at the expense of sensitivity. ChimPipe represents an advance in methods to quickly and reliably detect chimeric transcripts amongst the rapidly increasing volume of short read transcriptome data.

## Methods, results and discussion

In this section, we first present the ChimPipe method, then the ChimPipe benchmark, then the RT-PCR validation of ChimPipe predicted chimeras, and finally the application of ChimPipe to the search for common recurrent chimeras between human and mouse.

### The ChimPipe method

**Fig. 2:**
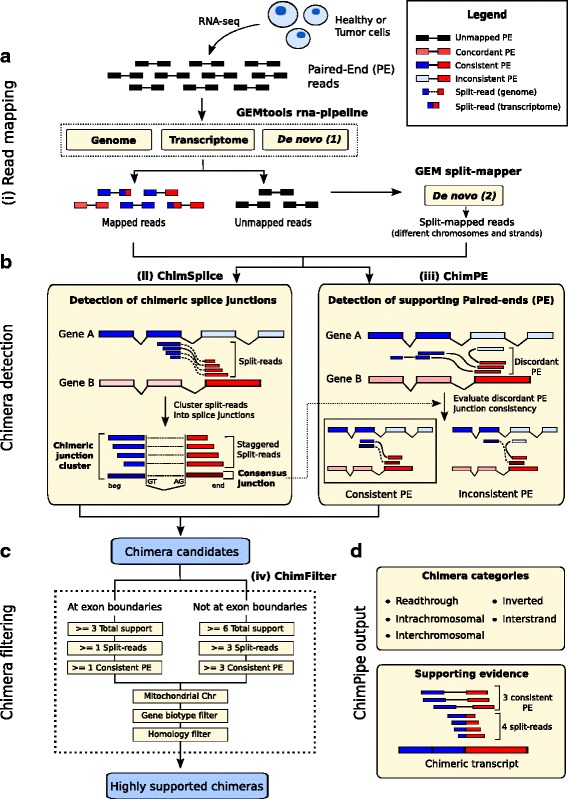
The ChimPipe method. **a** RNA-seq reads are first mapped to the genome and transcriptome using the GEMtools RNA-seq pipeline, and the reads that do not map this way are passed to the GEM RNA-mapper to get reads that split map to different chromosomes or strands. **b** The split-reads from these two mapping steps are then gathered and passed on to the *ChimSplice* module which derives consensus junctions associated to their expression calculated as the number of staggered split-reads supporting them. The *ChimPE* module can then associate each chimeric junction found by *ChimSplice* to their discordant PE reads, splitting them into the ones consistent and the ones inconsistent with the junction. **c** The *ChimFilter* module then applies a series of filters to the chimeric junctions obtained until this point in order to discard false positives, leading to **d** a set of reliable chimeric junctions to which it associates several pieces of information such as a category (readthrough, intrachromosomal, inverted, interstand, or interchromosomal), and the supporting evidence in terms of number of staggered split-reads and number of consistent PE reads, among others

The ChimPipe method is depicted in Fig. 2 and includes 4 consecutive steps:

(i) *Exhaustive paired-end and split read mapping with GEM*. The paired-end reads are initially mapped in 3 ways with the GEMtools RNA-seq pipeline (http://gemtools.github.io/docs/rna_pipeline.html): to the genome, to the transcriptome and *de novo*. Firstly, the reads are mapped to the genome with GEM [44], allowing up to 4% mismatches and indels. Secondly, the reads are mapped to the transcriptome with the same mapping parameters, the transcriptome being composed of all biologically valid combinations of exons within each gene (therefore also including annotated splice junctions). This transcriptome is built from the gene annotation and allows mapping of reads spanning exon to exon junctions that would not match to the reference genome due to the presence of introns. Thirdly, the reads are split-mapped to the genome with the GEM RNA mapper (http://algorithms.cnag.cat/wiki/The_GEM_library) to identify *de novo* splice junctions from unannotated transcripts. More precisely, reads are split into two segments of at least 15 base pair (bp) length, which are mapped independently to the genome. To reduce the amount of false positive mappings, only split-mappings with less than 4% mismatches or indels and harbouring extended consensus splice sites are further considered (GT+AG, GC+AG, ATATC+A. and GTATC+AT, with . meaning any nucleotide). To increase the mapping sensitivity, a second attempt is made by eroding a maximum of two bp towards the ends of each segment if no result is found. At this stage, segments can map to distant positions, but not to different chromosomes, different strands or reverse order. After that, genome, transcriptome and *de novo* mappings are merged and paired and those pairs mapping to more than 10 positions are set as unmapped. Finally, unmapped reads are remapped in a second *de novo* mapping with the GEM split-mapper (or RNA mapper). Reads are split-mapped to identify *bona fide* splice junctions connecting loci on different chromosomes, different strands and reverse order. Since this read split-mapping step is likely to generate more false positives than the initial ’normal’ read mapping step done with GEMtools, we decided to do the former in a more stringent way than the latter by not attempting to trim and remap the reads that did not map with the default parameters. Note that we chose GEM-based methods for mapping because these programs guarantee that all possible mappings of a read are reported given the input parameters.
(ii) *ChimSplice*. Read mapping is followed by candidate chimeric splice junction detection from split-mappings. The split-mapped reads are organized into clusters of reads spanning the same splice junction. The donor and acceptor splice sites are considered when building the clusters to guarantee that all of them are in the 5’ to 3’ orientation. This is very important to determine which are the upstream and downstream parent genes, and is particularly useful in case of unstranded RNA-seq data. Once the clusters have been generated, *ChimSplice* produces a consensus splice junction defined by the exact junction coordinates, the upstream coordinates of the upstream cluster, and the downstream coordinates of the downstream cluster. Additionally, each consensus junction is associated with the number of supporting split-reads and staggered split-reads. The term *staggered split-reads* refers to those reads spanning the same junction but mapping to different external positions and, as a consequence, producing a characteristic ladder-like pattern of reads across the junction (see Fig. 2 b). This pattern has been suggested to be specific to genuine chimeric transcripts, while false positives usually lack it [45]. This information is recorded and can be used to distinguish real from artefactual chimeras. Then, the consensus junctions are annotated. Each junction is compared to the annotated exons in order to determine its two parent genes. In case a junction side overlaps several exons from different genes, the one with a higher overlap is selected. Finally, splice junctions connecting exons from two different genes (chimeric junctions) are selected for downstream analyses.
(iii) *ChimPE*. Once chimeric junction candidates have been found using *ChimSplice*, *ChimPE* looks for further paired-end support for them (Fig. 2 b). Genome, transcriptome and *de novo* mappings are filtered to select only those PE reads with both mates mapped. Those PE reads are compared to annotated exons in the same way as described in (ii), and reads with both mates mapping to exons from different genes are identified (discordant PE reads). For each chimeric junction, discordant PE reads connecting their parent genes are then selected and their relative mapping position to the chimeric junction is evaluated. This is done in order to know whether the discordant PE reads support the existence of the chimeric junction (consistent PE) or if, on the other hand, they are incompatible with the chimeric junction (inconsistent PE). Inconsistent PE can be due to different reasons: they may come from a different chimeric RNA isoform than the one highlighted by *ChimSplice*, or from PE read misalignment, but they could also indicate a *ChimSplice* false positive. Finally, each chimeric junction is associated to the number of consistent and inconsistent PE reads, which can be used in the downstream *ChimFilter* filtering module to filter out artefactual chimeras.
(iv) *ChimFilter*. Chimeric junction candidates are filtered to produce a final set of more reliable chimeras. Firstly, based on the principle that false positives due to read misalignment would not be supported by both sources of evidence, ChimPipe requires a candidate chimera to be supported by both split-reads and consistent PE reads. Two different support based filtering schemes are applied depending on whether the chimeric junction involves annotated or novel splice sites. By default, chimeric junctions with annotated splice sites must be supported by at least one consistent PE read, one split-read and three total (consistent PE + split) reads, while those with novel splice sites have to be supported by at least three consistent PE reads, three split-reads and six total reads. Secondly, chimeric junctions involving genes either located on the mitochondrial chromosome or pseudogenic are filtered out as likely false positives due to mapping errors. Finally, chimeras between genes that share high exonic sequence similarity (at least 30 bp and 90% sequence identity) are also filtered out since their supporting reads are more prone to mis-alignments (Fig. 2 c). All these filtering parameters can be tuned.

The main ChimPipe output is a tabulated text file with header including the set of chimeric junctions after filtering, in which the first column is the junction identifier in ChimPipe format (donchr_donpos_donstr:accchr_accpos_accstr), and the other 34 columns are valuable pieces of information about it, such as its support in terms of number of staggered split-reads and consistent discordant paired-end reads, its type (*readthrough* (resp. *intrachromosomal*) if the two parts are on the same chromosome, same strand, expected genomic order and closer (resp. more distant) than 100 kilobase (kb), *inverted* if the two parts are on the same chromosome, same strand and unexpected genomic order, *interstrand* if the two parts are on the same chromosome but different strands and *interchromosomal* if the two parts are on different chromosomes), its two parent genes, its length and the list of its supporting reads (see Additional file 1: Table S1 for more details). ChimPipe also outputs a file with chimeric junctions before the filtering, and a file with the junctions that have been filtered out with information about the reason for this filtering (see ChimPipe user’s manual at http://chimpipe.readthedocs.io/en/latest/manual.html for more information). It has to be noted that ChimPipe can also start from already aligned reads (bam file) provided they include evidence of intra-chromosomal chimeric junctions, and that ChimPipe does not only output chimeric junctions but also a standard bam file (from step (i) of the pipeline) that can be used for more standard RNA-seq analyses such as differential gene expression or transcript reconstruction. Finally ChimPipe has been designed to require minimal information about the PE RNA-seq dataset on which it is run, since it guesses the Illumina quality offset, the strandedness, and the mate configuration in case of directional data. Note that ChimPipe’s documentation includes a tutorial and an example.

### Benchmark on simulated and cancer data

We evaluated ChimPipe and other state-of-the-art chimera detection tools, using two kinds of evaluation data: simulated data that we generated and real data from melanoma and breast cancer. The main advantages of simulated data are the inclusion of all kinds of chimeras (not only fusion genes) and the control over the chimeras expected to be found, therefore allowing a precise evaluation of the programs. Its main drawback, however, is the uncertainty about whether it captures the underlying complexity of real data. The drawback of real data, on the other hand, is its very limited number of validated cases, and the fact that most of them are fusion genes. Indeed neither does it allow to assess the programs’ precision, nor to extrapolate their results to non cancer data.

We developed ChimSim, a program to simulate chimeric transcripts from a gene annotation, a genome, and numbers of read-through, intra-chromosomal, inverted, interstrand and interchromosomal chimeric transcripts to create from the gene annotation (see Additional data section and Additional file 1: Supplementary methods). Using ChimSim on the the Gencode v19 protein-coding genes [46] and the hg19 genome, we generated a simulated dataset of 250 chimeric transcripts homogeneously distributed in the 5 chimera classes (50 from each class) (Additional file 2). Knowing that about 60% of transcripts from protein coding (pc) and long non-coding RNA (lncRNA) genes are usually expressed in a given condition [47], we sampled 60% of transcripts from Gencode v19 pc and lncRNA gene transcripts, totalling 101,961 transcripts (Additional file 2). Knowing that when a chimera is expressed, its parent genes are often also expressed [10], we added the parent transcripts of the 250 chimeras to the sampled transcripts, totalling 102,149 non chimeric transcripts (Additional file 2).

The 102,399 transcripts resulting from the union of the 250 chimeric transcripts and the 102,149 non-chimeric transcripts, were then passed on to the art_illumina program of the ART suite (version 2.3.7, [48]), to simulate Illumina non directional paired-end RNA-seq reads of 3 different lengths: 50bp, 76bp and 101bp, called PE50, PE76 and PE101 respectively. Several parameters were used in addition to read length and paired-endness, to make our simulated chimera data closer to real RNA-seq data, including insert size mean and standard deviation, read coverage and sequencing quality profile (see Additional file 1: Supplementary methods for details). The sequencing quality profile was learnt from real Illumina PE data of the same read length using the art_profiler_illumina program of the ART suite (version 2.3.7, Additional file 2 and Additional file 1: Supplementary methods). Using these parameters, ART generated 32.3, 21.1 and 15.7 million PE reads for the PE50, PE76 and PE101 respectively (Additional file 2 and Additional file 1: Supplementary methods). The benchmark was done for each read length separately.

For real data with experimentally validated chimeras, we used two previously published datasets: the leukemia/melanoma cancer study from Berger et al. ([25], that we call the Berger set), and the breast cancer study from Edgren et al. ([45], that we call the Edgren set). The Berger set was composed of the K562 chronic myelogenous leukemia cell line associated to two different insert size ranges, 300-400 bp and 400-600 bp, of the 501Mel melanoma cell line and of 5 melanoma patient-derived short-term cultures, and came with 14 RT-PCR validated fusion genes (Table 1). The Edgren set was composed of 4 breast cancer cell lines (of which two were associated to two different median insert sizes, 100bp and 200bp), and came with 27 RT-PCR validated fusion genes. For the Edgren set we used an additional 13 fusion genes that were found and RT-PCR-validated by a re-analysis of the Edgren et al. data by Kangaspeska et al. [49], totalling 40 fusion genes (Table 1). The benchmark was done for each library separately, but is provided for the pool of libraries of each dataset, for clarity reasons. Since the chimeras specifically targeted by the Berger and the Edgren studies were only fusion genes, the read-through events were removed from all programs’ predictions before running the benchmark.

**Table 1:** Cancer RNA-seq datasets used for benchmarking

Cancer dataset	Cell line	Tumor type	Number of validated	Number of validated	Number of different	SRA^a^ accession
			fusion genes	fusion junctions	libraries	codes
Berger	K-562	Leukemia	3	3	2	SRR018268,
						SRR0182689
	501 Mel	Melanoma	4	5	1	SRR018266
	M000216		1	1	1	SRR018259
	M000921		2	3	1	SRR018267
	M010403		1	1	1	SRR018265
	M980409		1	1	1	SRR018261
	M990802		2	2	1	SRR018260
	All	All	14	16	-	-
Edgren	KPL-4	Breast cancer	3	3	1	SRR064287
	MCF-7		6	8	1	SRR064286
	BT-474		21	25	2	SRR064438,
						SRR064439
	SK-BR-3		10	10	2	SRR064440,
						SRR064441
	All		40	46	1	-

This table indicates for each cancer dataset, its associated set of cell lines and corresponding tumor types, together with the number of RT-PCR validated fusion genes and junctions. Some fusion genes are associated to several fusion junctions

^a^SRA: Sequence Read Archive (http://www.ncbi.nlm.nih.gov/sra)

Since we wanted to do the evaluation both at the gene pair level and at the junction level, and since an RT-PCR validated fusion gene is merely a gene pair together with the cDNA sequence corresponding to its junction, we used the blat program [50] to align the cDNA sequences to the hg19 human genome, and further manually curated these alignments to obtain the exact chimeric junction coordinates for each fusion gene (see Additional file 1: Supplementary methods). This procedure resulted in 16 and 42 chimeric junctions for the Berger and Edgren sets respectively, indicating the presence of two different isoforms for one gene pair in each set (Additional file 1: Table S2).

The chimera detection programs that we chose to benchmark together with ChimPipe (version 0.9.3) were the following:

- FusionMap (version 8.0.2.32, [33])
- PRADA (version 1.2, [38])
- Chimerascan (version 0.4.5, [34])
- TopHatFusion (version 2.0.12, [32]).

We chose these programs because their method was published and for one of the following three reasons: (1) they were shown to have good results in several independent studies (for example FusionMap and Chimerascan) or (2) they were used in studies associated with gold-standard chimera RNA-seq datasets (for example PRADA in [25] and Chimerascan in [24]) or (3) they were extensively used by the community (for example TopHatFusion). Since these programs are optimized for human and for cancer, we used them with their default parameters for real data, and we adjusted their parameters to allow read-through detection for simulated data, when it was possible (see Additional file 1: Supplementary methods).

The evaluation measures used are the standard sensitivity and precision for simulated data, and the sensitivity and total number of predictions for real data. In addition, the evaluation was done at two levels: the gene pair level and the junction level (Additional file 1: Figure S1). For each of these two objects, gene pair and junction, we have a reference set (the objects to be predicted), and a predicted set for each program (the objects actually predicted by the program). We then define a true positive (TP) as an object present both in the reference and in the predicted set, a false positive (FP) as an object present in the predicted set but not in the reference set, and a false negative (FN) as an object present in the reference set but not in the predicted set. The sensitivity (Sn) is then the fraction of the reference objects that are correctly predicted, while the precision (Pr) is the fraction of the predicted objects that are correctly predicted. Since a high Sn can be easily obtained at the expense of a low Pr, and reciprocally, we use the F1score, which is the harmonic mean between Sn and Pr, as an additional measure. Note that in order for a predicted chimeric junction to be a TP, its coordinates must *exactly* match the coordinates of a reference chimeric junction (Additional file 1: Figure S1 and Supplementary methods).

**Fig. 3:**
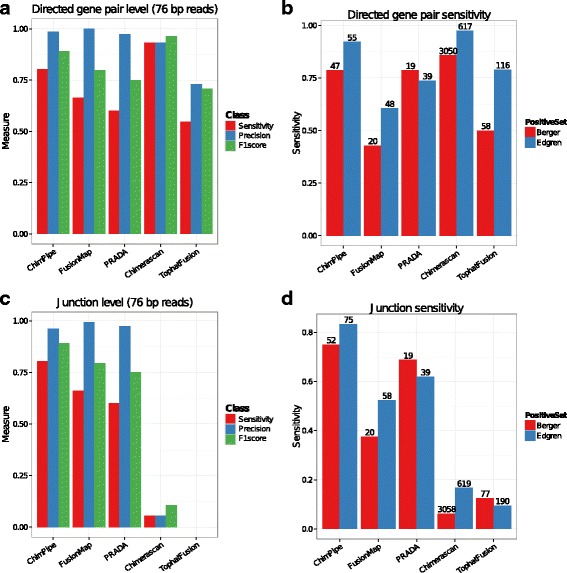
Benchmark results for 5 chimera detection programs on simulated (*left*) and on real (*right*) data. The sets of barplots on the top **a**, **b** indicate the programs’ performances at the gene pair level, while the sets of barplots at the bottom **c**, **d** indicate the programs’ performances at the junction level. For simulated data the provided measures are sensitivity (in *red*), precision (in *blue*), and F1score (in *green*), while for the two real datasets (Berger in *red* and Edgren in *blue*), the only provided measures are sensitivity (*bars*) and the total number of predictions (at the top of each bar). Here we show the results on PE76 simulated data, for the 250 simulated chimeric junctions (i.e. including read-through events). For the benchmark on real data, read-through events, i.e. junctions with a length smaller than 100kb when on the same chromosome, same strand and expected genomic order, were removed from the output of each program before the evaluation

The results at both the gene pair level and at the junction level for both the PE76 simulated data and the real data, are shown on Fig. 3 and Additional file 1: Table S3-S5 (similar results were observed for PE50 and PE101 except for FusionMap which is clearly better on PE76, see Additional file 1: Figure S2). At the gene pair level the top program on the simulated data is Chimerascan followed by ChimPipe, FusionMap, PRADA and finally TopHatFusion, with a generally quite high Pr for all programs but a Sn above 0.75 only for Chimerascan and ChimPipe. For real data, Chimerascan is still the top program in terms of Sn followed by ChimPipe, however its number of predicted gene pairs is 1 to 2 orders of magnitude higher than the one of ChimPipe. The trend for Sn on real data is similar to the one of simulated data, but the Edgren gene pairs seem to be easier to predict than the Berger gene pairs, with a higher Sn of the programs for the former. Note that PRADA is a program that also has a good compromise between Sn and number of predicted gene pairs on real data.

**Fig. 4:**
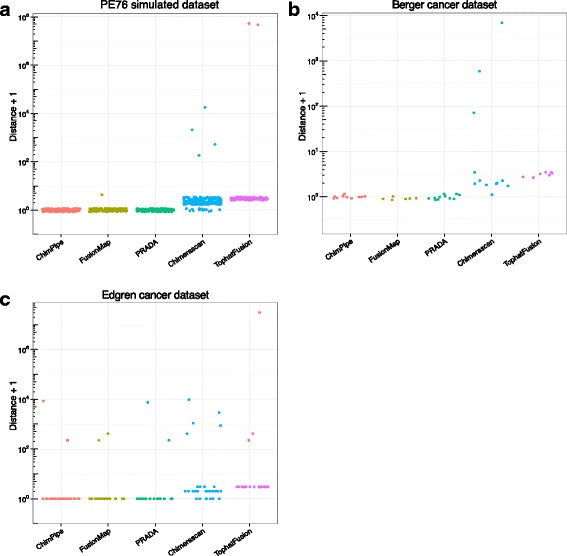
Distance between predicted and true junction. For the PE76 simulated set **a**, the Berger cancer dataset **b** and the Edgren cancer dataset **c**, and for each chimera detection program, the distance between the reference/true junction and the junction predicted by the program is plotted in log scale and using a pseudocount of 1 to avoid zero values. The distance between two junctions is defined as the sum of the distance between their donor/upstream/5’ splice sites and the distance between their acceptor/downstream/3’ splice sites

At the junction level, ChimPipe achieves the best results on both the simulated and the real data with a Sn around 0.8 and a Pr close to 1, and with a quite reasonable number of predicted junctions for real data (around 60). It is followed by PRADA and FusionMap, with PRADA behaving clearly better on real data. The performances of both Chimerascan and TopHatFusion are quite poor at the junction level, with TopHatFusion junctions most often shifted by 1 bp on each side (as if its coordinates were 0-based instead of 1-based), and Chimerascan junctions most often shifted by 1 bp on one or both sides, compared to true junctions. The fact that these programs do predict some junctions correctly (see Fig. 4 b-c), means that the incorrect junctions they predict cannot only be due to a different coordinate system.

Since some of the evaluated programs are not able to predict read-through events (PRADA), or happened to not detect any of them on simulated data (FusionMap and TopHatFusion), we also made an evaluation without read-through events on simulated data (Additional file 1: Figure S3). The effect was an overall improvement of the programs’ performances (except ChimPipe) but did not change the overall message above.

Since some programs have a quite different behaviour at the gene pair and at the junction level, we also computed for each program and each evaluation set, the average and standard deviation of the distance between the predicted and the true junction in case the gene pair was correctly predicted (Fig. 4 and Additional file 1: Table S6). It showed that ChimPipe, FusionMap and PRADA almost always provide the exact junction coordinates on simulated data and the Berger real set, while this is not the case for Chimerascan and TopHatFusion, with a worse behaviour for the latter on the simulated data and for the former on the Berger set. One can note that for simulated data, the distance between Chimerascan predicted and true junction tends to increase with read length (Additional file 1: Figure S4). Although the Edgren gene pairs seem easier to predict than the Berger gene pairs (as stated above), the junctions from the correctly predicted gene pairs seem more difficult to predict for the Edgren set than for the Berger set, since all the programs show a quite important average distance between predicted and true junction for the Edgren set (Fig. 4 and Additional file 1: Table S6). ChimPipe is second after FusionMap on the Edgren set but also has many more true positives on this set. Since when ChimPipe detects the correct gene pair it also detects the correct junction both for the simulated data and for the Berger cancer data, we think that the most likely explanation for this difficulty in finding the true junction for some Edgren cases is the fact that the mRNA isoform represented by the RT-PCR sequence is not the same as the one sequenced with RNA-seq.

**Fig. 5:**
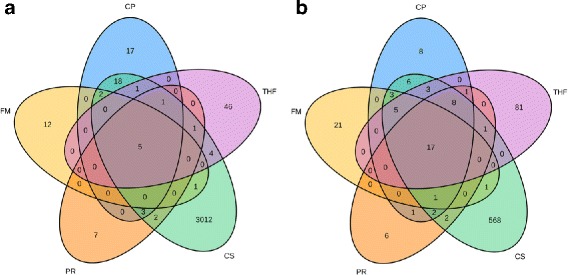
Chimeric gene pairs predicted by the 5 programs on the two real datasets. Intersection between chimeric gene pairs predicted by the 5 programs on the Berger set **a** and on the Edgren set **b** are represented as Venn diagrams. In general gene pairs predicted by all 5 programs are few compared to the gene pairs predicted by a single program, and we expect that the higher the number of programs predicting a gene pair the more reliable the gene pair. Chimerascan and TophaFusion are the programs that predict more gene pairs predicted by no other program, while PRADA, Chimpipe and FusionMap are the programs with less such gene pairs. CP: ChimPipe, FM: FusionMap, PR: PRADA, CS: Chimerascan, THF: TopHatFusion

Although real data does not allow to compute precision or false positive rate, we expect the number of programs predicting a given chimera to be correlated to the likelihood of this chimera to be a TP. We computed the intersection between the gene pairs predicted by each program on each of the two real sets (Berger and Edgren) (Fig. 5), and saw that PRADA, ChimPipe and FusionMap predicted fewer unique gene pairs, while TopHatFusion and Chimerascan predicted many unique gene pairs, consistent with the previous benchmark results (Fig. 3). We also confirmed that a gene pair predicted by at least 2 programs was more likely to be real since 26% (respectively 65%) of the gene pairs predicted by 2 programs on the Berger (resp. Edgren) set are TP (i.e. validated by RT-PCR), while only 0% (respectively 1%) of the ones predicted by 1 program are TP.

Regarding implementation, while some programs require a single step to predict chimeras (apart from the genome and/or transcriptome indexing), which is the case for ChimPipe, FusionMap and Chimerascan, some other programs require many different successive steps to obtain them, making the whole process more cumbersome. This is the case for PRADA which requires 3 steps (mapping script making + mapping + chimera prediction) and for TopHatFusion which requires 2 steps (mapping + filtering). The maximum virtual memory and wallclock time needed by each program (run with 4 threads) on the PE76 simulated data are provided in Table 2. The program that clearly needs the least resources is FusionMap with 11.7 Gb of RAM and less than half an hour of running time, followed by Chimerascan with 4.8 Gb of RAM and 8.2 h of running time, then PRADA with 35.5 Gb of RAM and 4.5 h of running time, then ChimPipe with 34.5 Gb of RAM and 10.1 h of running time, and finally TopHatFusion which requires 62.2 Gb of RAM and 18 h of running time.

**Table 2:** Resources needed by the programs run with 4 threads

Program	Maximum RAM used (in Gb)	Cumulative wallclock time (in hours)	Number of commands to execute
ChimPipe	34.5	10.1	1
FusionMap	11.7	0.4	1
PRADA	35.5	4.5	3 (make mapping script, mapping, compute fusion)
Chimerascan	4.8	8.2	1
TophatFusion	62.2	18^a^	2 (mapping, filtering)

This table indicates the computing resources needed by each program to process the PE76 simulated data, as well as the number of commands needed to produce the final result

^a^9 h for mapping and 9 h for filtering (27.5 h for the 3 simulated sets (50bp, 76bp and 101bp) at the same time)

### Detection and validation of novel chimeras

In order to survey the human chimera landscape more extensively, ChimPipe was run on 106 ENCODE CSHL PE RNA-seq experiments from 15 human cell lines, 3 RNA fractions (polyadenylated, non-polyadenylated, total) and 6 cell compartments (whole cell, cytosol, nucleus, chromatin, nucleolus, nucleoplasm) ([47] and Additional file 1: Table S7). At stringent settings (10 supporting staggered split-reads and 5 discordant paired-end reads in at least one experiment), we found a total of 1195 chimeric junctions over all experiments. Of these, 525 had each of their two ends falling in a unique different protein coding gene, and 142 were either expressed recurrently (at least 1 supporting read in at least 11 out of the 15 cell lines) or very highly and specifically (at least 100 total reads in a single cell line). We then only considered the 137 read-through and intrachromosomal chimeric junctions from this set (Additional file 3).

Four of these chimeric junctions were chosen for RT-PCR plus Sanger sequencing validation. Two of them were selected from the recurrently expressed class (RPL38-TTYH2 and UBA2-WTIP), and two of them from the very highly and specifically expressed class (PICALM-SYTL2 and C16orf62-IQCK) (Table 3). Primers were designed to perform RT-PCR on cDNA (to test for the RNA chimera) as well as PCR on genomic DNA, to assess whether the chimeras could originate from genomic rearrangements (Additional file 1: Figure S5 and Tables S8-S9). Out of those 4 cases, all showed evidence of the two parent gene mRNAs (except one, SYTL2, but this could be due to a low expression level of this gene), and 3 showed the additional presence of the chimeric RNA (Additional file 1: Figure S6 and Supplementary methods). These 3 chimeric junctions present at the RNA level, were not present at the DNA level and therefore cannot originate from genomic rearrangements (Additional file 1: Figure S7 and Supplementary methods). We cloned and sequenced these 3 chimeras (UBA2-WTIP, PICALM-SYTL2 and RPL38-TTYH2) (Additional file 1: Figure S8, Additional file 4, Fig. 6 b for UBA2-WTIP, and Additional file 1: Supplementary methods). Given that the genes they connect are on the same chromosome, strand and close to each other, these 3 chimeras are likely to originate from read-through events (even if trans-splicing cannot be totally excluded).

**Fig. 6:**
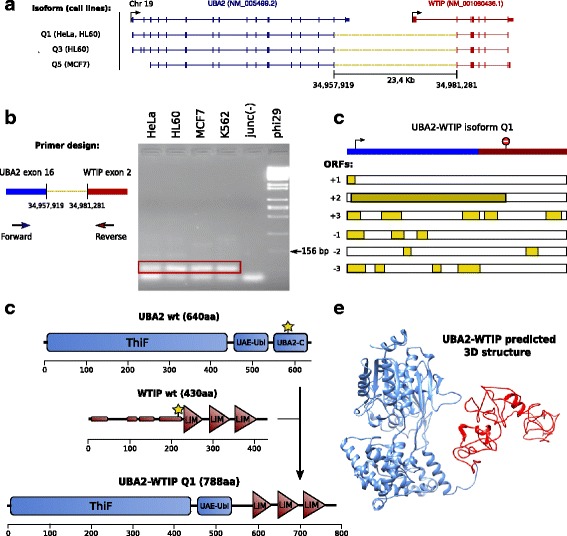
UBA2-WTIP chimeric transcript isoforms. **a** Experimentally validated UBA2-WTIP chimeric transcript isoforms. (*Top*) UBA2 and WTIP parent transcripts according to RefSeq version 74. Coding and UTR exonic sequences are displayed as *thick* and *thin* boxes, respectively, and introns as lines. The genomic strand of the transcripts is represented as an arrow on the 5’ end (*Bottom*) Chimeric RNAs with chimeric splice junctions are depicted as *yellow dashed lines*. On the left, list of cancer cell lines where each isoform was validated **b** UBA2-WTIP chimeric splice junction validation (*Left*) Primer design for validating the chimeric junction through RT-PCR plus Sanger sequencing. (*Right*) Chimeric junction validation in 4 different cell lines. The 72 bp amplicons proving the expression of the chimeric RNAs are highlighted in *red*. **c** UBA2-WTIP Q1 isoform protein coding potential. (*Top*) UBA2 and WTIP annotated start and stop codons represented over the transcript sequence. (*Bottom*) ORFs in the six possible frames. The selected ORF from the UBA2 annotated start codon to the WTIP annotated stop codon is highlighted in *dark yellow*. **d** Putative chimeric protein encoded by the UBA2-WTIP Q1 isoform. (*Top*) UBA2 and WTIP wild-type proteins. The exact position of the two protein breakpoints is indicated by *yellow stars*. Protein domains are depicted as boxes and triangles over the protein sequences. Thin boxes on the WTIP protein sequence correspond to low complexity regions. The x axis shows the amino acid position along the protein sequence. (*Bottom*) Putative UBA2-WTIP chimeric protein. Full-length domains are represented over the protein sequence. **e** The predicted 3D structure of the UBA2-WTIP chimeric protein as modelled by Phyre2 [51]. The chimeric protein part derived from UBA2 is depicted in blue and the one derived from WTIP in *red*

**Table 3:** ENCODE RNA-seq chimeric junctions sent to RT-PCR validation

Chimeric junction identifier	Gene A	Gene B	SK-N-SH_RA:4	AG04450:4	MCF-7:4	H1-hESC:7	GM12878:9	A549:4	HUVEC:11	NHEK:13	HepG2:11	BJ:2	NHLF:4	HSMM:4	K562:16	HeLa-S3:11	HMEC:2
chr19_34957919	UBA2	WTIP	7.3:0.5	3:0.3	3.5:0.8	2.3:0.7	3.1:1.6	12.5:0.8	4.2:1.5	2.2:0.2	2.5:0.2	3:0	2.3:1.3	4:5.3	7.1:1.2	6.6:1.7	6:2.5
_+:chr19_																	
34981281_+																	
chr11_85685751_-:chr11_85469157_-	PICALM	SYTL2	0:0	4.5:0	5.3:90.8	0:0	1.2:0.2	0.8:0	0.7:0	0.9:0	0.2:0.1	1.5:0	0:0	1.8:0.3	2.3:0.1	0.3:0	0:0
chr17_72200329_	RPL38	TTYH2	2.8:0	0.3:0	0.5:0	0.3:0.4	4.3:5.7	0.5:0	0.9:0.7	0.5:0	0.6:1.4	0.5:0	0.3:0	0.3:0.5	0.3:0.7	0.1:0	0:0
+:chr17_72218624_+																	
chr16_19603196_+	C16orf62	IQCK	0:0	0:0	33.8:65.8	0:0	0:0	0:0	0:0	0:0	0:0	0:0	0:0	0:0	0:0	0:0	0:0
:chr16_19867809_+																	

This table lists the 4 chimeric junctions derived from the 106 ENCODE RNA-seq experiment, that we selected for RT-PCR validation (the successful ones are the three first ones). For each junction, its identifier (junction coordinates in ChimPipe format), the name of its 5’ and 3’ genes, and its expression in terms of number of staggered split-reads and discordant paired-end reads in each of the 15 ENCODE cell lines (separated by :), are provided. Since each cell line is associated to several RNA-seq experiments (number indicated in the header after the : sign), we provide the average number of staggered split-reads and the average number of discordant paired end reads across all experiments of a cell line

It has been suggested that the generation of chimeric transcripts and their translation into chimeric proteins may serve to generate novel proteins with altered functions [1, 10]. Therefore, we assessed the protein-coding potential of the 3 validated chimeric junctions. For each chimeric junction, we reconstructed the theoretical chimeric transcript structures by combining the RefSeq reference mRNAs for the 5’ and 3’ parent genes compatible with the junction and searched for Open Reading Frames (ORFs) in the six possible translational frames with the NCBI ORF Finder (http://www.ncbi.nlm.nih.gov/gorf/gorf.html). One case out of the 3 (UBA2-WTIP), which has already been reported [20], maintained the frame of the two parent genes, UBA2 and WTIP, while the other two, PICALM-SYTL2 and RPL38-TTYH2, did not. Interestingly, this chimera is recurrently expressed in 72 out of the 106 experiments, which include the 15 cell lines, the 3 RNA fractions and 5 out of the 6 cell compartments (cell, cytosol, nucleus, nucleoplasm and chromatin) (Table 3 and Additional file 3). Additional RT-PCR and Sanger sequencing was therefore performed on UBA2-WTIP, giving rise to 3 novel complete transcript structures (Fig. 6 a, Additional file 5), of which the longest one (Q1), was more deeply analysed here. This complete chimeric transcript has an ORF from UBA2 to WTIP annotated start and stop codon respectively (Fig. 6 c). Thus, if translated it would give rise to a chimeric protein including the two most N-terminal domains of the 5’ parent protein UBA2 (ThiF and UAE_Ubl domains) and the three most C-terminal domains of the 3’ parent protein WTIP (LIM domains), therefore only skipping the UBA2_C domain of the UBA2 protein and the proline-rich N-terminal domain of the WTIP protein (Fig. 6 d). Finally, Phyre2 structural prediction analysis [51] of this chimera is able to model 97% of its residues at more than 90% confidence. This analysis suggests that the chimeric protein part derived from UBA2 can fold into a 3D structure with 100% confidence and 96% identity to UBA2 wild-type fold. On the other hand, the WTIP protein part can fold with 99% confidence and 29% identity to LDB1, a member of the same family of LIM domain-containing proteins as WTIP. These data are consistent with the hypothesis that the UBA2-WTIP protein may at least partially retain the biochemical activity of both parent proteins, leading to a protein with an altered function (Fig. 6 e).

We further investigated the putative role of this chimeric protein containing the combination of domains from UBA2 and WTIP wild-type proteins. UBA2 is part of the SUMOylation machinery, which post-translationally modifies and regulates a large number of proteins with important roles in diverse cellular processes, including regulation of transcription, chromatin structure, and DNA repair [52]. More precisely, it associates with the Aos1 protein to produce the SUMO-activating enzyme (E1), a heterodimer that mediates the activation of ubiquitin-related modifier (SUMO) molecules and their transference to the SUMO-conjugating enzyme (E2), which post-translationally modifies a target protein through the binding of SUMO [53]. On the other hand, WTIP belongs to a subset of LIM-domain containing proteins, which are involved in focal and cell-cell adhesion. These interact with other proteins through their LIM domains, whose sequence specifies a double zinc-finger structure capable of high-affinity binding to a wide variety of protein targets [54]. Based on this, we hypothesize that the combination of UBA2 SUMOylation domain and WTIP protein binding LIM domains could lead to a chimeric protein with altered SUMOylation activity. This protein may induce the SUMOylation machinery to post-translationally modify and regulate novel targets, due to the interaction of its LIM-domains with novel proteins.

Finally, each one of the two other validated chimeras, PICALM-SYTL2 and RPL38-TTYH2, gave rise to one novel (although incompletely identified) transcript structure with a premature stop codon before the last splice junction, leading us to hypothesize that they are degraded through nonsense-mediated mRNA decay [55]. However, it is important to note that these chimeric junctions are supported by a very high number of reads (Table 3), suggesting that the chimeric transcripts are highly expressed, and possibly functional.

### Common recurrent chimeras between human and mouse

In order to find common, and therefore potentially evolutionary conserved, chimeras between human and mouse, we applied ChimPipe to human and mouse ENCODE RNA-seq data [56]. These data consist of 2 bioreplicates of 18 human cell lines and 30 mouse tissues, from which long polyadenylated RNA was extracted and deeply sequenced (at least 100 million PE reads). We applied ChimPipe to each bioreplicate separately, and asked each reported junction to be supported by at least one split-read and one discordant PE read (see Additional file 1: Supplementary methods). We then gathered all reported junctions within each species, and found a total of 9339 chimeric junctions in human and 6447 chimeric junctions in mouse. In order to discard chimeras derived from genomic rearrangements, we also required the chimeras to be recurrent, i.e. detected in at least 2 samples (see Additional file 1: Supplementary methods). This filtering reduced the number of chimeras to 3567 in human and 3284 in mouse, corresponding to 2572 and 2372 gene pairs respectively. A common chimera between human and mouse was then defined by the combined presence of a chimera connecting genes A and B in human, and of a chimera connecting the ortholog of gene A and the ortholog of gene B in mouse. Requiring the connected genes to belong to the set of 15,736 one-to-one human-mouse orthologs [57], we obtained 1596 chimeras in human and 1610 chimeras in mouse, corresponding to 1119 and 1096 gene pairs respectively, and the subset of those that were common between the 2 species were 211 junctions in human and 197 junctions in mouse, corresponding to 131 gene pairs (Additional file 6). The large majority of these common chimeras connected genes on the same chromosome, same strand, expected genomic order and relatively close to each other (median distance: 10kb), pointing to *read-through* as the main underlying mechanism. We also found that many tens of such chimeras were detected in more than 10 samples in one species or the other, confirming the existence and wide expression of *read-through* chimeras in non cancerous cells [3, 4, 12, 20, 30]. We also found that not only the gene pairs were common between human and mouse, but also the chimeric junctions. Indeed from the 131 common gene pairs, 40 were supported by at least one chimeric junction where the 2 splice sites both in human and in mouse were in our set of orthologous splice sites [56], of which 31 (78%) were supported by at least one chimeric junction connecting orthologous splice sites in the 2 species. The cell lines and tissues with more chimeric junctions in the 2 species were related to brain (SK-N-SN and SK-N-SN_RA in human and embryonic central nervous system in mouse) (Additional file 6).

## Conclusions

We have presented ChimPipe, a novel method for the accurate detection of chimeras from PE RNA-seq data, based on the independent use of discordant PE reads and split-reads. In addition to fusion genes and trans-splicing events, ChimPipe is able to detect read-through events, which is now recognized as the most prevalent class of real chimeras in both normal and tumour tissues [20, 29, 30]. ChimPipe is general enough to be able to work on any eukaryotic species with a genome and an annotation available. This allows to study chimera evolution but also to investigate the impact of chimeras on individuals from species on which we have more control than human (for example livestock). ChimPipe can also predict several isoforms per gene pair and the exact chimeric junction coordinates, which are essential for chimeric transcript reconstruction and downstream biological validation.

ChimPipe is easy to run since it only requires a genome, a gene annotation and a pair of RNA-seq fastq files (once the indexing of the genome and transcriptome have been done), and guesses many other things such as the directionality, the mate configuration and the Illumina offset quality. For advanced users, many parameters, such as expression threshold or parent gene sequence similarity threshold, can be tuned. ChimPipe provides both a complete and a filtered set of chimeric junctions, with additional information about them, such as chimera category, expression support and the list of reads supporting the junction (Additional file 1: Table S1). In addition to chimeric junctions, ChimPipe provides a standard bam file obtained from the GEMtools RNA pipeline (step (i) of Fig. 2), that can be used for downstream analyses such as differential gene expression or transcript reconstruction.

Benchmarking of ChimPipe together with four state-of-the art chimera detection tools on both simulated and real data, showed ChimPipe to have a very good precision (close to 1), and to be the second most sensitive program (Sn of ≈0.8), therefore showing a very good balance between sensitivity and precision. Additionally ChimPipe’s performances on simulated and real data are comparable, and not much impacted by read length. ChimPipe’s performances are also similar at the gene pair and at the junction level, which is not the case for all programs since they tend to predict gene pairs better than junctions (see Fig. 3). It has to be noted that ChimPipe needs non negligible computer resources to achieve these results, since it requires ≈30 Gb of RAM and half a day to run with 4 threads, on the PE76 simulated data (21 million PE reads).

The application of ChimPipe to 106 ENCODE PE RNA-seq samples allowed the detection of 137 highly reliable chimeras, of which 4 were chosen for RT-PCR validation, and of which 3 were indeed validated and further cloned and sequenced. The UBA2-WTIP chimera additionally preserved the frame of the 2 parent genes UBA2 and WTIP, and was therefore completely sequenced, leading to 3 completely novel transcript structures. If translated these 3 novel transcripts would lead to a chimeric protein with the ThiF and the UAE-Ubf domains from the UBA2 protein and with the 3 LIM domains from the WTIP protein. We hypothesize that this protein may induce the SUMOylation machinery to post-translationally modify and regulate novel targets, due to the interaction of its LIM-domains with novel proteins.

The application of ChimPipe to 36 human and 60 mouse ENCODE PE RNA-seq experiments also allowed the identification of 131 recurrent chimeras common to both species, and therefore potentially conserved. Although their large majority connect adjacent genes and should originate from read-through events, some cases are also distant or located on different chromosomes. Tens of them are detected in more than 10 samples.

Despite these advantages, ChimPipe could be improved in at least 2 aspects: (1) it could provide all the chimeric transcripts compatible with the chimeric junction (module for which we already have a tested code) as additional information, (2) it could be made more robust by being reimplemented in a pipeline specific language such as nextflow (http://www.nextflow.io/).

Finally it has to be noted that our contribution goes beyond the ChimPipe program, since we provide two additional programs: (1) a chimera simulator program, called ChimSim (https://github.com/Chimera-tools/ChimSim), and (2) a chimera benchmark program, called ChimBench (https://github.com/Chimera-tools/ChimBench). We also provide new realistic simulated data, as well as junction coordinates for validated fusion genes from 2 extensively used gold-standard chimera datasets [25, 45]. We think that, in addition to ChimPipe, both these programs and these data can be very useful in future chimera detection assessments.

## Additional files

Additional file 1 Supplementary Tables, Figures and Methods (PDF 717 kb)

Additional file 2 Simulated data (TAR.GZ 7.4Gb)

Additional file 3 ENCODE read-through and intra-chromosomal chimeras (TSV 102kb)

Additional file 4 Junction sequences for 3 RT-PCR validated chimeras (DOCX 4.39 kb)

Additional file 5 UBA2-WTIP transcript isoform sequences (DOCX 142kb)

Additional file 6 Common chimeras between human and mouse (TSV 52kb)

## Abbreviations

bp: Base pair
cDNA: Complementary DNA
EST: Expressed sequence tag
Gb: Gigabyte
kb: Kilobase
mRNA: Messenger RNA
PE: Paired-end
RT-PCR: Reverse transcriptase polymerase chain reaction
RAM: Random access memory
3D: Three dimensional

## References

[1] Gingeras TR (2009). Implications of chimaeric non-co-linear transcripts. Nature.

[2] Mitelman F, Johansson B, Mertens F (2007). The impact of translocations and gene fusions on cancer causation. Nat Rev Cancer.

[3] Akiva P, Toporik A, Edelheit S, Peretz Y, Diber A, Shemesh R (2006). Transcription-mediated gene fusion in the human genome. Genome Res.

[4] Parra G, Reymond A, Dabbouseh N, Dermitzakis ET, Castelo R, Thomson TM (2006). Tandem chimerism as a means to increase protein complexity in the human genome. Genome Res.

[5] Unneberg P, Claverie JM (2007). Tentative mapping of transcription-induced interchromosomal interaction using chimeric EST and mRNA data. PLoS ONE.

[6] Birney E, Stamatoyannopoulos JA, Dutta A, Guigó R, Gingeras TR, Margulies EH (2007). Identification and analysis of functional elements in 1% of the human genome by the ENCODE pilot project. Nature.

[7] Denoeud F, Kapranov P, Ucla C, Frankish A, Castelo R, Drenkow J (2007). Prominent use of distal 5 t́ranscription start sites and discovery of a large number of additional exons in ENCODE regions. Genome Res.

[8] Djebali S, Kapranov P, Foissac S, Lagarde J, Reymond A, Ucla C (2008). Efficient targeted transcript discovery via array-based normalization of RACE libraries. Nat Methods.

[9] Djebali S, Lagarde J, Kapranov P, Lacroix V, Borel C, Mudge JM (2012). Evidence for transcript networks composed of chimeric RNAs in human cells. PLoS ONE.

[10] Frenkel-Morgenstern M, Lacroix V, Ezkurdia I, Levin Y, Gabashvili A, Prilusky J (2012). Chimeras taking shape: potential functions of proteins encoded by chimeric RNA transcripts. Genome Res.

[11] Hernández-Torres F, Rastrojo A, Aguado B (2013). Intron retention and transcript chimerism conserved across mammals: Ly6g5b and Csnk2b-Ly6g5b as examples. BMC Genomics.

[12] Prakash T, Sharma VK, Adati N, Ozawa R, Kumar N, Nishida Y (2010). Expression of conjoined genes: another mechanism for gene regulation in eukaryotes. PloS ONE.

[13] Li H, Wang J, Mor G, Sklar J (2008). A neoplastic gene fusion mimics trans-splicing of RNAs in normal human cells. Science.

[14] Kannan K, Wang L, Wang J, Ittmann MM, Li W, Yen L (2011). Recurrent chimeric RNAs enriched in human prostate cancer identified by deep sequencing. Proc Natl Acad Sci.

[15] Wu CS, Yu CY, Chuang CY, Hsiao M, Kao CF, Kuo HC (2014). Integrative transcriptome sequencing identifies trans-splicing events with important roles in human embryonic stem cell pluripotency. Genome Res.

[16] Rickman DS, Pflueger D, Moss B, VanDoren VE, Chen CX, de la Taille A (2009). SLC45A3-ELK4 is a novel and frequent erythroblast transformation–specific fusion transcript in prostate cancer. Cancer Res.

[17] Li X, Zhao L, Jiang H, Wang W (2009). Short homologous sequences are strongly associated with the generation of chimeric RNAs in eukaryotes. J Mol Evol.

[18] Cocquet J, Chong A, Zhang G, Veitia RA (2006). Reverse transcriptase template switching and false alternative transcripts. Genomics.

[19] Houseley J, Tollervey D (2010). Apparent non-canonical trans-splicing is generated by reverse transcriptase in vitro. PLoS ONE.

[20] Greger L, Su J, Rung J, Ferreira PG, Lappalainen T, Dermitzakis ET (2014). Tandem RNA chimeras contribute to transcriptome diversity in human population and are associated with intronic genetic variants. PloS ONE.

[21] Nowell PC (1960). A minute chromosome in human granulocytic leukemia. Science.

[22] Tomlins SA, Rhodes DR, Perner S, Dhanasekaran SM, Mehra R, Sun XW (2005). Recurrent fusion of TMPRSS2 and ETS transcription factor genes in prostate cancer. Science.

[23] Tomlins SA, Laxman B, Dhanasekaran SM, Helgeson BE, Cao X, Morris DS (2007). Distinct classes of chromosomal rearrangements create oncogenic ETS gene fusions in prostate cancer. Nature.

[24] Maher CA, Palanisamy N, Brenner JC, Cao X, Kalyana-Sundaram S, Luo S (2009). Chimeric transcript discovery by paired-end transcriptome sequencing. Proc Natl Acad Sci.

[25] Berger MF, Levin JZ, Vijayendran K, Sivachenko A, Adiconis X, Maguire J (2010). Integrative analysis of the melanoma transcriptome. Genome Res.

[26] Zhang Y, Gong M, Yuan H, Park HG, Frierson HF, Li H (2012). Chimeric transcript generated by cis-splicing of adjacent genes regulates prostate cancer cell proliferation. Cancer Discov.

[27] Pflueger D, Mittmann C, Dehler S, Rubin MA, Moch H, Schraml P (2015). Functional characterization of BC039389-GATM and KLK4-KRSP1 chimeric read-through transcripts which are up-regulated in renal cell cancer. BMC Genomics.

[28] Grosso AR, Leite AP, Carvalho S, Matos MR, Martins FB, Vítor AC (2015). Pervasive transcription read-through promotes aberrant expression of oncogenes and RNA chimeras in renal carcinoma. Elife.

[29] Ferreira PG, Jares P, Rico D, Gómez-López G, Martínez-Trillos A, Villamor N (2014). Transcriptome characterization by RNA sequencing identifies a major molecular and clinical subdivision in chronic lymphocytic leukemia. Genome Res.

[30] Babiceanu M, Qin F, Xie Z, Jia Y, Lopez K, Janus N (2016). Recurrent chimeric fusion RNAs in non-cancer tissues and cells. Nucleic Acids Res.

[31] Sboner A, Habegger L, Pflueger D, Terry S, Chen DZ, Rozowsky JS (2010). FusionSeq: a modular framework for finding gene fusions by analyzing paired-end RNA-sequencing data. Genome Biol.

[32] Kim D, Salzberg SL (2011). TopHat-Fusion: an algorithm for discovery of novel fusion transcripts. Genome Biol.

[33] Ge H, Liu K, Juan T, Fang F, Newman M, Hoeck W (2011). FusionMap: detecting fusion genes from next-generation sequencing data at base-pair resolution. Bioinformatics.

[34] Iyer MK, Chinnaiyan AM, Maher CA (2011). ChimeraScan: a tool for identifying chimeric transcription in sequencing data. Bioinformatics.

[35] McPherson A, Hormozdiari F, Zayed A, Giuliany R, Ha G, Sun MG (2011). deFuse: an algorithm for gene fusion discovery in tumor RNA-Seq data. PLoS Comput Biol.

[36] Benelli M, Pescucci C, Marseglia G, Severgnini M, Torricelli F, Magi A (2012). Discovering chimeric transcripts in paired-end RNA-seq data by using EricScript. Bioinformatics.

[37] Jia W, Qiu K, He M, Song P, Zhou Q, Zhou F (2013). SOAPfuse: an algorithm for identifying fusion transcripts from paired-end RNA-Seq data. Genome Biol.

[38] Torres-García W, Zheng S, Sivachenko A, Vegesna R, Wang Q, Yao R (2014). PRADA: pipeline for RNA sequencing data analysis. Bioinformatics.

[39] Fernandez-Cuesta L, Sun R, Menon R, George J, Lorenz S, Meza-Zepeda LA (2015). Identification of novel fusion genes in lung cancer using breakpoint assembly of transcriptome sequencing data. Genome Biol.

[40] Wang Q, Xia J, Jia P, Pao W, Zhao Z (2013). Application of next generation sequencing to human gene fusion detection: computational tools, features and perspectives. Brief Bioinform.

[41] Beccuti M, Carrara M, Cordero F, Donatelli S, Calogero RA (2013). The structure of state-of-art gene fusion-finder algorithms. Genome Bioinformatics.

[42] Carrara M, Beccuti M, Cavallo F, Donatelli S, Lazzarato F, Cordero F (2013). State of art fusion-finder algorithms are suitable to detect transcription-induced chimeras in normal tissues?. BMC Bioinformatics.

[43] Carrara M, Beccuti M, Lazzarato F, Cavallo F, Cordero F, Donatelli S (2013). State-of-the-art fusion-finder algorithms sensitivity and specificity. BioMed Res Int.

[44] Marco-Sola S, Sammeth M, Guigó R, Ribeca P (2012). The GEM mapper: fast, accurate and versatile alignment by filtration. Nat Methods.

[45] Edgren H, Murumagi A, Kangaspeska S, Nicorici D, Hongisto V, Kleivi K (2011). Identification of fusion genes in breast cancer by paired-end RNA-sequencing. Genome Biol.

[46] Harrow J, Frankish A, Gonzalez JM, Tapanari E, Diekhans M, Kokocinski F (2012). GENCODE: the reference human genome annotation for The ENCODE Project. Genome Res.

[47] Djebali S, Davis CA, Merkel A, Dobin A, Lassmann T, Mortazavi A (2012). Landscape of transcription in human cells. Nature.

[48] Huang W, Li L, Myers JR, Marth GT (2012). ART: a next-generation sequencing read simulator. Bioinformatics.

[49] Kangaspeska S, Hultsch S, Edgren H, Nicorici D, Murumägi A, Kallioniemi O (2012). Reanalysis of RNA-sequencing data reveals several additional fusion genes with multiple isoforms. PloS ONE.

[50] Kent WJ (2002). BLAT: the BLAST-like alignment tool. Genome Res.

[51] Kelley LA, Mezulis S, Yates CM, Wass MN, Sternberg MJ (2015). The Phyre2 web portal for protein modeling, prediction and analysis. Nat Protoc.

[52] Gill G (2004). SUMO and ubiquitin in the nucleus: different functions, similar mechanisms?. Genes Dev.

[53] Johnson ES (2004). Protein modification by SUMO. Annu Rev Biochem.

[54] Dawid IB, Breen JJ, Toyama R (1998). LIM domains: multiple roles as adapters and functional modifiers in protein interactions. Trends Genet.

[55] Brogna S, Wen J (2009). Nonsense-mediated mRNA decay (NMD) mechanisms. Nat Struct Mol Biol.

[56] Pervouchine DD, Djebali S, Breschi A, Davis CA, Barja PP, Dobin A (2015). Enhanced transcriptome maps from multiple mouse tissues reveal evolutionary constraint in gene expression. Nat Commun.

[57] Yue F, Cheng Y, Breschi A, Vierstra J, Wu W, Ryba T (2014). A comparative encyclopedia of DNA elements in the mouse genome. Nature.

