# Supplementary material for: ChimPipe: accurate detection of fusion genes and transcription-induced chimeras from RNA-seq data
Source: BMC Genomics. 2017 Jan 3;18:7. doi: 10.1186/s12864-016-3404-9 (PMC5209911; doi:10.1186/s12864-016-3404-9)
Supplement: Additional file 1 — Supplementary Tables, Figures and Methods (PDF 717 kb) [file 12864_2016_3404_MOESM1_ESM.pdf]

ChimPipe: Accurate detection of fusion genes and transcription-induced chimeras from RNA-seq data

Bernardo Rodríguez-Martín, Emilio Palumbo, Santiago Marco-Sola, Thasso Griebel, Paolo Ribeca, Graciela Alonso, Alberto Rastrojo, Begoña Aguado, Roderic Guigó, Sarah Djebali

Supplementary Tables, Figures and Methods

ChimPipe filtered chimeric junction file description

Name of the field	Meaning of the field	Example of value
juncCoord	Chimeric junction identifier	chr12_112375965_-:chr12_49727016_-
type	Chimeric junction category	intrachromosomal
filtered	Whether the junction was filtered out	0
reason	Reason for filtering it out	na
nbTotal(spanning+consistent)	No reads supporting the junction	25
nbSpanningReads	No split-reads supporting the junction	15
nbStaggered	No staggered split-reads supporting the junction	14
percStaggered	Percentage of split-reads that are staggered	93.3333
nbMulti	No multi-mapped split-reads supporting the junction	3
percMulti	% split-reads that are multi-mapped	20
nbConsistentPE	No consistent discordant PE supporting the junction	10
nbInconsistentPE	No inconsistent discordant PE supporting the junction	0
percInconsistentPE	% discordant PE that are inconsistent with the junction	0
overlapA	% overlap between 5' part and annotated exons	94.2308
overlapB	% overlap between 3' part and annotated exons	98.9899
distExonBoundaryA	Distance between donor site and annotated donor	0
distExonBoundaryB	Distance between acc. site and annotated acc.	0
blastAlignLen	Max. length of BLAST alignment between gene pair	na
blastAlignSim	Max. % sim. of BLAST alignment	na
donorSS	Donor 2bp splice site sequence	GT
acceptorSS	Acceptor 2bp splice site sequence	AG
beg	5' part most 5' coordinate	112376016
end	3' part most 3' coordinate	49726918
sameChrStr	Whether 2 parts are on same chr. and strand	1
okGxOrder	Whether 2 parts are in expected gx order	1
dist	Distance between 2 parts if SameChrStr & okGxOrder	62648949
gnIdsA	List of 5' parent gene ids	ENSG00000198270.8
gnIdsB	List of 3' parent gene ids	ENSG00000186897.3
gnNamesA	List of 5' parent gene names	TMEM116
gnNamesB	List of 3' parent gene names	C1QL4
gnTypesA	List of 5’ parent gene biotypes	protein_coding
gnTypesB	List of 3’ parent gene biotypes	protein_coding
juncSpanningReadsIds	List of supporting split-read ids	ENST00000549537.2-ENST00000334221.3-214/1,...,ENST00000549537.2-ENST00000334221.3-228/2,
consistentPEIds	List of consistent discordant PE read ids	ENST00000549537.2-ENST00000334221.3-196,...,ENST00000549537.2-ENST00000334221.3-222,
inconsistentPEIds	List of inconsistent discordant PE read ids	na

Table S1. This table explains the different fields/columns present in the filtered chimeric junction file output by ChimPipe (tsv file). For each field the name of the field (as indicated in the header), the meaning of the field and an example of value of the field are indicated.

Chimeric junction coordinates for the cancer RT-PCR validated fusion genes

Dataset	Fusion Gene	Chimeric junction number	Chimeric junction identifier in ChimPipe format	Sample
Berger	BCR:ABL1	1	chr22_23632600_+:chr9_133729451_+	K-562
	BAG6:SLC44A4	1	chr6_31619433_-:chr6_31833561_-	K-562
	NUP214:XKR3	1	chr9_134074402_+:chr22_17288973_-	K-562
	CCT3:C1orf61	1	chr1_156294763_-:chr1_156377767_-	501Mel
	CCT3:C1orf61	2	chr1_156294763_-:chr1_156374393_-	501Mel
	GNA12:SHANK2	1	chr7_2834562_-:chr11_70742672_-	501Mel
	SLC12A7:AAMDC	1	chr5_1085347_-:chr11_77580768_+	501Mel
	PARP1:MIXL1	1	chr1_226576357_-:chr1_226413208_+	501Mel
	KCTD2:ARHGEF12	1	chr17_73055700_+:chr11_120351969_+	M000216
	TMEM8B:TLN1	1	chr9_35835215_+:chr9_35717783_-	M000921
	RECK:ALX3	1	chr9_36108161_+:chr1_110604185_-	M000921
	RECK:ALX3	2	chr9_36105280_+:chr1_110604185_-	M000921
	SCAMP2:WDR72	1	chr15_75137814_-:chr15_53998271_-	M010403
	GCN1L1:PLA2G1B	1	chr12_120582110_-:chr12_120763823_-	M980409
	ANKHD1:CYSTM1	1	chr5_139781858_+:chr5_139622890_+	M990802
	RB1:ITM2B	1	chr13_48881542_+:chr13_48830313_+	M990802
Edgren	BSG:NFIX	1	chr19_580782_+:chr19_13135835_+	KPL-4
	PPP1R12A:SEPT10	1	chr12_80211174_-:chr2_110343415_-	KPL-4
	NOTCH1:NUP214	1	chr9_139438476_-:chr9_134062676_+	KPL-4
	DIDO1:TTI1	1	chr20_61569148_-:chr20_36634799_-	BT-474
	RPS6KB1:SNF8	1	chr17_57970686_+:chr17_47021337_-	BT-474
	VAPB:IKZF3	1	chr20_56964573_+:chr17_37934020_-	BT-474
	ACACA:STAC2	1	chr17_35479453_-:chr17_37374426_-	BT-474
	ZMYND8:CEP250	1	chr20_45852970_-:chr20_34078463_+	BT-474
	RAB22A:MYO9B	1	chr20_56886178_+:chr19_17256207_+	BT-474
	SKA2:MYO19	1	chr17_57232492_-:chr17_34863763_-	BT-474
	STARD3:DOK5	1	chr17_37793484_+:chr20_53259997_+	BT-474
	LAMP1:MCF2L	1	chr13_113951810_+:chr13_113718618_+	BT-474
	GLB1:CMTM7	1	chr3_33055548_-:chr3_32483332_+	BT-474
	CPNE1:PI3	1	chr20_34243124_-:chr20_43804502_+	BT-474
	THRA:AC090627.1	1	chr17_38243106_+:chr17_46384693_+	BT-474
	THRA:AC090627.1	2	chr17_38243106_+:chr17_46371709_+	BT-474
	TOB1:SYNRG	1	chr17_48943419_-:chr17_35880751_-	BT-474
	AHCTF1:NAAA	1	chr1_247094880_-:chr4_76846964_-	BT-474
	MED1:STXBP4	1	chr17_37607291_-:chr17_53218671_+	BT-474
	MED13:BCAS3	1	chr17_60129898_-:chr17_59469338_+	BT-474
	MED13:BCAS3	2	chr17_60129898_-:chr17_59465979_+	BT-474
	MED1:ACSF2	1	chr17_37595418_-:chr17_48548389_+	BT-474
	TRPC4AP:MRPL45	1	chr20_33665849_-:chr17_36478009_+	BT-474
	TRPC4AP:MRPL45	2	chr20_33665849_-:chr17_36476502_+	BT-474
	TRPC4AP:MRPL45	3	chr20_33665849_-:chr17_36474586_+	BT-474
	STX16:RAE1	1	chr20_57227143_+:chr20_55929088_+	BT-474
	USP32:MED1	1	chr17_58422842_-:chr17_37604157_-	BT-474
	PIP4K2B:RAD51C	1	chr17_36933940_-:chr17_56809845_+	BT-474
	RARA:PKIA	1	chr17_38465538_+:chr8_79485046_+	SK-BR-3
	TATDN1:GSDMB	1	chr8_125551266_-:chr17_38066177_-	SK-BR-3
	ANKHD1:PCDH1	1	chr5_139825560_+:chr5_141234001_-	SK-BR-3
	CCDC85C:SETD3	1	chr14_100002352_-:chr14_99880271_-	SK-BR-3
	SUMF1:LRRFIP2	1	chr3_4418014_-:chr3_37170640_-	SK-BR-3
	CYTH1:EIF3H	1	chr17_76778284_-:chr8_117768036_-	SK-BR-3
	DHX35:ITCH	1	chr20_37597857_+:chr20_32957200_+	SK-BR-3
	TBC1D31:ZNF704	1	chr8_124096580_+:chr8_81733850_-	SK-BR-3
	BCAS4:BCAS3	1	chr20_49411710_+:chr17_59445688_+	MCF-7
	ARFGEF2:SULF2	1	chr20_47538547_+:chr20_46365686_-	MCF-7
	RPS6KB1:VMP1	1	chr17_57992064_+:chr17_57917129_+	MCF-7
	GCN1L1:MSI1	1	chr12_120628101_-:chr12_120785317_-	MCF-7
	AC099850.1:VMP1	1	chr17_57184952_+:chr17_57915656_+	MCF-7
	SMARCA4:CARM1	1	chr19_11097269_+:chr19_11015627_+	MCF-7

Table S2. This table provides for each validated fusion gene in each of the 2 cancer datasets, the chimeric junction number, its location on the genome in ChimPipe format and the sample where it was validated.

Benchmark results on simulated data

Program	Simulated dataset	Gene pair level								Junction level							
		Reference	Predicted	TP	FN	Sensitivity	FP	Precision	F1score	Reference	Predicted	TP	FN	Sensitivity	FP	Precision	F1score
ChimPipe			205	199	51	0.80	6	0.97	0.89		206	199	51	0.80	7	0.97	0.89
FusionMap			97	97	153	0.39	0	1.00	0.56		97	97	153	0.39	0	1.00	0.56
PRADA	PE50	250	160	155	95	0.62	5	0.97	0.77	250	160	155	95	0.62	5	0.97	0.77
Chimerascan			255	236	14	0.94	19	0.93	0.97		255	14	236	0.06	241	0.05	0.11
TophatFusion			200	143	107	0.57	57	0.72	0.73		200	0	250	0.00	200	0.00	0.00
ChimPipe			204	201	49	0.80	3	0.99	0.89		209	201	49	0.80	8	0.96	0.89
FusionMap			166	166	84	0.66	0	1.00	0.80		166	165	85	0.66	1	0.99	0.80
PRADA	PE76	250	154	150	100	0.60	4	0.97	0.75	250	154	150	100	0.60	4	0.97	0.75
Chimerascan			250	233	17	0.93	17	0.93	0.96		250	14	236	0.06	236	0.06	0.11
TophatFusion			188	137	113	0.55	51	0.73	0.71		188	0	250	0.00	188	0.00	0.00
ChimPipe			195	189	61	0.76	6	0.97	0.86		202	188	62	0.75	14	0.93	0.86
FusionMap			123	123	127	0.49	0	1.00	0.66		123	123	127	0.49	0	1.00	0.66
PRADA	PE101	250	144	141	109	0.56	3	0.98	0.72	250	144	141	109	0.56	3	0.98	0.72
Chimerascan			247	226	24	0.90	21	0.91	0.95		247	14	236	0.06	233	0.06	0.11
TophatFusion			170	132	118	0.53	38	0.78	0.69		170	0	250	0.00	170	0.00	0.00

Table S3. This table provides the numbers related to the benchmark of the 5 chimera detection programs on the 3 simulated datasets (PE50, PE76, PE101), both at the gene pair and at the junction level. Reference: number of reference objects (gene pairs or junctions); Predicted: number of predicted objects; TP: number of correctly predicted objects; FN: number of reference objects that are not predicted; Sensitivity: fraction of reference objects correctly predicted; FP: number of predicted objects not in the reference; Precision: fraction of predicted objects that are in the reference; F1score: harmonic mean between sentivity and precision.

Benchmark results on simulated data (no read-through)

Program	Simulated dataset	Gene pair level								Junction level							
		Reference	Predicted	TP	FN	Sensitivity	FP	Precision	F1score	Reference	Predicted	TP	FN	Sensitivity	FP	Precision	F1score
ChimPipe	PE50	202	164	160	42	0.79	4	0.98	0.88	202	165	160	42	0.79	5	0.97	0.88
FusionMap			97	97	105	0.48	0	1.00	0.65		97	97	105	0.48	0	1.00	0.65
PRADA			160	155	47	0.77	5	0.97	0.87		160	155	47	0.77	5	0.97	0.87
Chimerascan			214	195	7	0.97	19	0.91	0.98		214	14	188	0.07	200	0.07	0.13
TophatFusion			200	143	59	0.71	57	0.72	0.83		200	0	202	0.00	200	0.00	0.00
ChimPipe	PE76	202	168	165	37	0.82	3	0.98	0.90	202	173	165	37	0.82	8	0.95	0.90
FusionMap			165	165	37	0.82	0	1.00	0.90		165	164	38	0.81	1	0.99	0.90
PRADA			154	150	52	0.74	4	0.97	0.85		154	150	52	0.74	4	0.97	0.85
Chimerascan			208	191	11	0.95	17	0.92	0.97		208	14	188	0.07	194	0.07	0.13
TophatFusion			188	137	65	0.68	51	0.73	0.81		188	0	202	0.00	188	0.00	0.00
ChimPipe	PE101	202	157	152	50	0.75	5	0.97	0.86	202	164	151	51	0.75	13	0.92	0.86
FusionMap			122	122	80	0.60	0	1.00	0.75		122	122	80	0.60	0	1.00	0.75
PRADA			144	141	61	0.70	3	0.98	0.82		144	141	61	0.70	3	0.98	0.82
Chimerascan			206	185	17	0.92	21	0.90	0.96		206	14	188	0.07	192	0.07	0.13
TophatFusion			170	132	70	0.65	38	0.78	0.79		170	0	202	0.00	170	0.00	0.00

Table S4. This table provides the numbers related to the benchmark of the 5 chimera detection programs on the 3 simulated datasets (PE50, PE76, PE101) when excluding read-through events, both at the gene pair and at the junction level. Reference: number of reference objects (gene pairs or junctions); Predicted: number of predicted objects; TP: number of correctly predicted objects; FN: number of reference objects that are not predicted; Sensitivity: fraction of reference objects correctly predicted; FP: number of predicted objects not in the reference; Precision: fraction of predicted objects that are in the reference; F1score: harmonic mean between sentivity and precision.

Benchmark results on the cancer datasets

Program	Cancer dataset	Gene pair level					Junction level				
		Reference	Predicted	TP	FN	Sensitivity	Reference	Predicted	TP	FN	Sensitivity
ChimPipe			47	11	3	0.79		52	12	4	0.75
FusionMap			20	6	14	0.43		20	6	10	0.38
PRADA	Berger	14	19	11	8	0.79	16	19	11	5	0.69
Chimerascan			3050	12	3038	0.86		3058	1	15	0.06
TophatFusion			58	7	51	0.50		77	2	14	0.13
ChimPipe			55	35	20	0.92		75	35	7	0.83
FusionMap			48	23	25	0.61		58	22	20	0.52
PRADA	Edgren	38	39	28	11	0.74	42	39	26	16	0.62
Chimerascan			617	37	580	0.97		619	7	35	0.17
TophatFusion			116	30	86	0.79		190	4	38	0.10

Table S5. This table provides the numbers related to the benchmark of the 5 chimera detection programs on the 2 cancer datasets (Berger and Edgren), both at the gene pair and at the junction level. Reference: number of reference objects (gene pairs or junctions); Predicted: number of predicted objects; TP: number of correctly predicted objects; FN: number of reference objects that are not predicted; Sensitivity: fraction of reference objects correctly predicted.

Distance between predicted and true junction

Program	PE76 simulated data				Berger cancer data				Edgren cancer data			
	# reference junctions	# predicted junctions from correctly predicted gene pairs	distance between predicted and true junction		# reference junctions	# predicted junctions from correctly predicted gene pairs	distance between predicted and true junction		# reference junctions	# predicted junctions from correctly predicted gene pairs	distance between predicted and true junction	
			AVG	STD			AVG	STD			AVG	STD
ChimPipe		201	0.0	0.0		11	0.0	0.0		35	247.6	1426.7
FusionMap		166	0.0	0.3		6	0.0	0.0		23	27.6	95.7
PRADA	250	150	0.0	0.0	16	11	0.0	0.0	42	28	274.4	1408.9
Chimerascan		233	99.2	1305.6		12	592.2	1866.8		37	403.0	1639.4
TophatFusion		137	753605.0	6343310.0		7	2.0	0.0		30	1015780.0	5563540.0

Table S6. This table provides for each benchmark dataset (PE76 simulated, Berger and Edgren) and each program, the number of reference chimeric junctions, the number of predicted junctions corresponding to correctly predicted gene pairs and the average (AVG) and standard deviation (STD) of the distance between the predicted and the true junction for those. This distance is computed as the sum of the distance between the predicted and the true donor and the distance between the predicted and the true acceptor.

ENCODE CSHL PE RNA-seq data

geo_accession_number	labExpId	cell_line	rna_fraction	cell_compartment	bioreplicate_number
GSM767854	LID9005	A549	longNonPolyA	cell	1
GSM767854	LID9006	A549	longNonPolyA	cell	2
GSM758564	LID8963	A549	longPolyA	cell	1
GSM758564	LID8964	A549	longPolyA	cell	2
GSM765396	LID9001	AG04450	longNonPolyA	cell	1
GSM765396	LID9002	AG04450	longNonPolyA	cell	2
GSM758561	LID8965	AG04450	longPolyA	cell	1
GSM758561	LID8966	AG04450	longPolyA	cell	2
GSM767855	LID9007	BJ	longNonPolyA	cell	1
GSM767855	LID9008	BJ	longNonPolyA	cell	2
GSM758572	LID8661	GM12878	longNonPolyA	cell	1
GSM758572	LID8662	GM12878	longNonPolyA	cell	2
GSM767852	LID18547	GM12878	longNonPolyA	cytosol	1
GSM767853	LID9197	GM12878	longNonPolyA	nucleus	1
GSM767853	LID9198	GM12878	longNonPolyA	nucleus	2
GSM758559	LID16629	GM12878	longPolyA	cell	1
GSM758559	LID16630	GM12878	longPolyA	cell	2
GSM758560	LID8467	GM12878	longPolyA	cytosol	1
GSM758560	LID8468	GM12878	longPolyA	cytosol	2
GSM758573	LID8663	H1-hESC	longNonPolyA	cell	1
GSM758573	LID8664	H1-hESC	longNonPolyA	cell	2
GSM767842	LID18549	H1-hESC	longNonPolyA	cytosol	2
GSM758566	LID8461	H1-hESC	longPolyA	cell	1
GSM758566	LID8462	H1-hESC	longPolyA	cell	2
GSM758570	LID8536	H1-hESC	longPolyA	cytosol	2
GSM758574	LID8558	H1-hESC	longPolyA	nucleus	2
GSM767847	LID8790	HeLa-S3	longNonPolyA	cell	1
GSM767847	LID8791	HeLa-S3	longNonPolyA	cell	2
GSM767838	LID18551	HeLa-S3	longNonPolyA	cytosol	2
GSM767848	LID9200	HeLa-S3	longNonPolyA	nucleus	1
GSM767848	LID9201	HeLa-S3	longNonPolyA	nucleus	2
GSM765402	LID16633	HeLa-S3	longPolyA	cell	1
GSM765402	LID16634	HeLa-S3	longPolyA	cell	2
GSM765404	LID8469	HeLa-S3	longPolyA	cytosol	1
GSM765404	LID8470	HeLa-S3	longPolyA	cytosol	2
GSM765403	LID8559	HeLa-S3	longPolyA	nucleus	1
GSM765403	LID8560	HeLa-S3	longPolyA	nucleus	2
GSM758567	LID8792	HepG2	longNonPolyA	cell	1
GSM758567	LID8793	HepG2	longNonPolyA	cell	2
GSM767840	LID18552	HepG2	longNonPolyA	cytosol	1
GSM767840	LID18553	HepG2	longNonPolyA	cytosol	2
GSM767850	LID9202	HepG2	longNonPolyA	nucleus	1
GSM767850	LID9203	HepG2	longNonPolyA	nucleus	2
GSM758575	LID16635	HepG2	longPolyA	cell	1
GSM758575	LID16636	HepG2	longPolyA	cell	2
GSM758576	LID8471	HepG2	longPolyA	cytosol	1
GSM758576	LID8472	HepG2	longPolyA	cytosol	2
GSM758568	LID8534	HepG2	longPolyA	nucleus	1
GSM765397	LID8831	HMEC	longNonPolyA	cell	1

GSM758571	LID8695	HMEC	longPolyA	cell	1
GSM765391	LID8826	HSMM	longNonPolyA	cell	1
GSM765391	LID8827	HSMM	longNonPolyA	cell	2
GSM758578	LID8710	HSMM	longPolyA	cell	1
GSM758578	LID8711	HSMM	longPolyA	cell	2
GSM767856	LID8788	HUVEC	longNonPolyA	cell	1
GSM767856	LID8789	HUVEC	longNonPolyA	cell	2
GSM767839	LID18556	HUVEC	longNonPolyA	cytosol	3
GSM767857	LID9206	HUVEC	longNonPolyA	nucleus	3
GSM767857	LID9207	HUVEC	longNonPolyA	nucleus	4
GSM758563	LID8463	HUVEC	longPolyA	cell	1
GSM758563	LID8464	HUVEC	longPolyA	cell	2
GSM758569	LID8688	HUVEC	longPolyA	cytosol	3
GSM758569	LID8689	HUVEC	longPolyA	cytosol	4
GSM758565	LID8690	HUVEC	longPolyA	nucleus	3
GSM758565	LID8691	HUVEC	longPolyA	nucleus	4
GSM758577	LID8659	K562	longNonPolyA	cell	1
GSM758577	LID8660	K562	longNonPolyA	cell	2
GSM767844	LID9195	K562	longNonPolyA	nucleus	1
GSM767844	LID9196	K562	longNonPolyA	nucleus	2
GSM765405	LID16627	K562	longPolyA	cell	1
GSM765405	LID16628	K562	longPolyA	cell	2
GSM840137	LID8465	K562	longPolyA	cytosol	1
GSM840137	LID8466	K562	longPolyA	cytosol	2
GSM765387	LID8556	K562	longPolyA	nucleus	1
GSM765387	LID8557	K562	longPolyA	nucleus	2
GSM765392	LID9185	K562	total	chromatin	3
GSM765392	LID9186	K562	total	chromatin	4
GSM765393	LID9187	K562	total	nucleolus	3
GSM765393	LID9188	K562	total	nucleolus	4
GSM765390	LID9189	K562	total	nucleoplasm	3
GSM765390	LID9190	K562	total	nucleoplasm	4
GSM767851	LID8824	MCF-7	longNonPolyA	cell	1
GSM767851	LID8825	MCF-7	longNonPolyA	cell	2
GSM765388	LID8686	MCF-7	longPolyA	cell	1
GSM765388	LID8687	MCF-7	longPolyA	cell	2
GSM765398	LID8665	NHEK	longNonPolyA	cell	1
GSM765398	LID8666	NHEK	longNonPolyA	cell	2
GSM765398	LID8830	NHEK	longNonPolyA	cell	5
GSM767843	LID18554	NHEK	longNonPolyA	cytosol	3
GSM767846	LID9204	NHEK	longNonPolyA	nucleus	3
GSM767846	LID9205	NHEK	longNonPolyA	nucleus	4
GSM765401	LID16631	NHEK	longPolyA	cell	1
GSM765401	LID16632	NHEK	longPolyA	cell	2
GSM765401	LID8694	NHEK	longPolyA	cell	5
GSM765400	LID8795	NHEK	longPolyA	cytosol	3
GSM765400	LID8796	NHEK	longPolyA	cytosol	4
GSM765399	LID8797	NHEK	longPolyA	nucleus	3
GSM765399	LID8798	NHEK	longPolyA	nucleus	4
GSM765389	LID8828	NHLF	longNonPolyA	cell	1
GSM765389	LID8829	NHLF	longNonPolyA	cell	2
GSM765394	LID8692	NHLF	longPolyA	cell	1
GSM765394	LID8701	NHLF	longPolyA	cell	2

GSM767845	LID9003	SK-N-SH_RA	longNonPolyA	cell	1
GSM767845	LID9011	SK-N-SH_RA	longNonPolyA	cell	2
GSM765395	LID8967	SK-N-SH_RA	longPolyA	cell	1
GSM765395	LID8968	SK-N-SH_RA	longPolyA	cell	2

Table S7. This table provides metadata information about the 106 ENCODE CSHL PE RNA-seq experiments used by ChimPipe to produce the 137 chimeras on which 4 were selected for RT-PCR validation.

RT-PCR primer sequences

Primer name	Sequence (5'→3')
PICALMjF	CCTCCACAAATGGGAAGTGT
SYTL2jR	CTCTCTTCTTCGGCCCTCTT
PICALMmRNAR	TGCTGCTTGAGCACTTGTCT
SYTL2mRNAF	GGGCCAGAAGACAAGAGAGA
PICALMgDNAR	TCCTTTCCCTCCAAATAATCAA
SYTL2gDNAF	TGCCTCTCACTGTCTGGTGT
C16orf62jF	GTTCTACCCAAGCAAATTTGTCCTTAT
IQCKjR	AATTTTCATCTTGGCTGCAGGT
C16orf62mRNAR	GGCTATCCACACACATGGAA
C16orf62gDNAR	CCCAATTCTTGACAAAACGAA
IQCKmRNAF	TTCAAGAACTGCGTCAGTGG
IQCKgDNAF	GGTTTTGGTGCAACTGACCT
RPL38_j_F	AAGGACTTCCTGCTCACAGC
TTYH2_j_R	CAAGGAAGATGAGGTTCAGG
RPL38_mRNA_R	TGCCTTCTCTTTGTCAGTGATG
RPL36_gDNA_R	ATGAAACCACTGCTCCCAGA
TTYH2_mRNA_F	GACTACATCGCTCCCTGGTG
TTYH2_gDNA_F	CTTGGAAGGATGGACGAGAG
SLC44A4_j_F	ACACTGGGTCATTGGCATTT
BAG6_j_R	CCCCCACAATAAAGGTACGA
SLC44A4_mRNA_R	ACTTTGTCCAGGACGACCAC
BAG6_mRNA_F	ACTATCGGGGAAACGGAAGT
SLC44A4_gDNA_R	GAGGAAGGCTCATGTTTGGT
BAG6_gDNA_F	TGTGTGTGTGTGCAGGAGAA
UBA_j_F	CAATGGCAGTGATGATGGAG
WTIP_j_R	GTAGATGCCAAGCCCACACT
UBA_mRNA_R	CCTCTTGCGGCTTCTCTCTT
WTIP_mRNA_F	CGCCTACGCTGACTTCCTC
UBA2_gDNA_R	ACCAAACCAATACACACCTCT
WTIP_gDNA_F	ACCCTGATGGTGCCCATGTT
ELOVL7_j_F	GTGGTTTGGGAACATTCCAT
DEPDC1B_j_R	CCAGTACACTGACAAAAGCATCA
ELOVL7_mRNA_R	AATGATGCACGCAAAGACTG
DEPDC1B_mRNA_F	AACTTCCTCATTGGGTGCTG
ELOVL7_gDNA_R	TCAAAGTTGCATTCCCTCAA
DEPDC1B_gDNA_F	TTTTGCTCAACTCTATGACTGTAGG
Q1extFwd	CCTCAAGAATCTCGTGCTCA
Q1_3extRvs	CAGGCACAGACGGTGAATAA
Q3extFwd	TGCAGTTTTACCCGAAAGCTA
Q1intFwd	GGTTTCTCCCACATCGACCT
Q1_3intRvs	TGCTCAGAGCTCAGTGACG

Table S8. This table provides the sequences of the primers used for the RT-PCR validation of the ENCODE chimeras.

Usage of RT-PCR primer pairs

Primer pair		Purpose	Target gene
PICALMjF	PICALMmRNAR	Individual gene in cDNA	PICALM
PICALMjF	PICALMgDNAR	Individual gene in gDNA	PICALM
SYTL2gDNAF	SYTL2jR	Individual gene in gDNA	SYTL2
SYTL2mRNAF	SYTL2jR	Individual gene in cDNA	SYTL2
PICALMjF	SYTL2jR	Chimeric junction	---
C16orf62jF	C16orf62mRNAR	Individual gene in cDNA	C16orf62
C16orf62jF	C16orf62gDNAR	Individual gene in gDNA	C16orf62
IQCKmRNAF	IQCKjR	Individual gene in cDNA	IQCK
IQCKgDNAF	IQCKjR	Individual gene in gDNA	IQCK
C16orf62jF	IQCKjR	Chimeric junction	---
RPL38_j_F	RPL38_mRNA_R	Individual gene in cDNA	RPL38
RPL38_j_F	RPL38_gDNA_R	Individual gene in gDNA	RPL38
TTYH2_mRNA_F	TTYH2_j_R	Individual gene in cDNA	TTYH2
TTYH2_gDNA_F	TTYH2_j_R	Individual gene in gDNA	TTYH2
RPL38_j_F	TTYH2_j_R	Chimeric junction	---
UBA_j_F	UBA_mRNA_R	Individual gene in cDNA	UBA
UBA_j_F	UBA2_gDNA_R	Individual gene in gDNA	UBA
WTIP_mRNA_F	WTIP_j_R	Individual gene in cDNA	WTIP
WTIP_gDNA_F	WTIP_j_R	Individual gene in gDNA	WTIP
UBA_j_F	WTIP_j_R	Chimeric junction	---
SLC44A4_j_F	SLC44A4_mRNA_R	Individual gene in cDNA	SLC44A4
SLC44A4_j_F	SLC44A4_gDNA_R	Individual gene in gDNA	SLC44A4
BAG6_mRNA_F	BAG6_j_R	Individual gene in cDNA	BAG6
BAG6_gDNA_F	BAG6_j_R	Individual gene in gDNA	BAG6
SLC44A4_j_F	BAG6_j_R	Chimeric junction	---
ELOVL7_j_F	ELOVL7_mRNA_R	Individual gene in cDNA	ELOVL7
ELOVL7_j_F	ELOVL7_gDNA_R	Individual gene in gDNA	ELOVL7
DEPDC1B_mRNA_F	DEPDC1B_j_R	Individual gene in cDNA	DEPDC1B
DEPDC1B_gDNA_F	DEPDC1B_j_R	Individual gene in gDNA	DEPDC1B

Table S9. This table provides for each primer pair used for RT-PCR validation (see Table S8), its purpose (individual gene in completementary or genomic DNA, chimeric junction) and in case it was targetting a gene, the name of this gene.

Figure S1. Two evaluation levels: gene pair level and junction level. On top is one reference junction with its associated gene pair (gene A, gene B). At the bottom are two predicted junctions of which the first one exactly corresponds to the reference junction, and is therefore considered both a junction level and a gene pair level true positive (TP). The second junction does not exactly correspond to the reference junction, and is therefore not considered a junction level TP, however since its first part overlaps an exon of gene A and its second part an exon of gene B, it is considered a gene pair level TP.

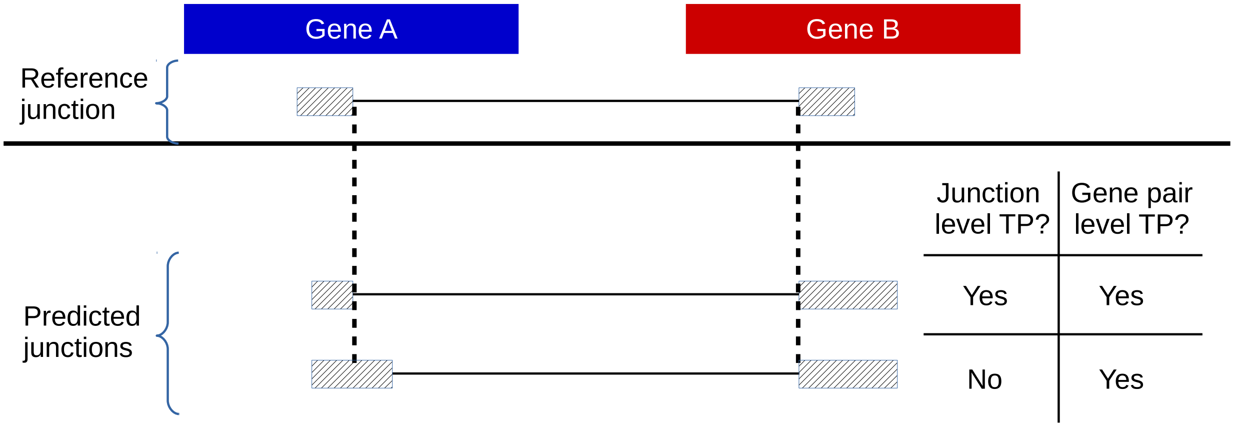

Figure S3. Benchmark results when excluding read-through events for simulated data. This is the same as figure 3 but excluding read-through events when benchmarking the programs on simulated data. Note that figures S3B and S3D are the same as figures 3B and 3D, and are present here for the purpose of comparison between results on simulated and real data.

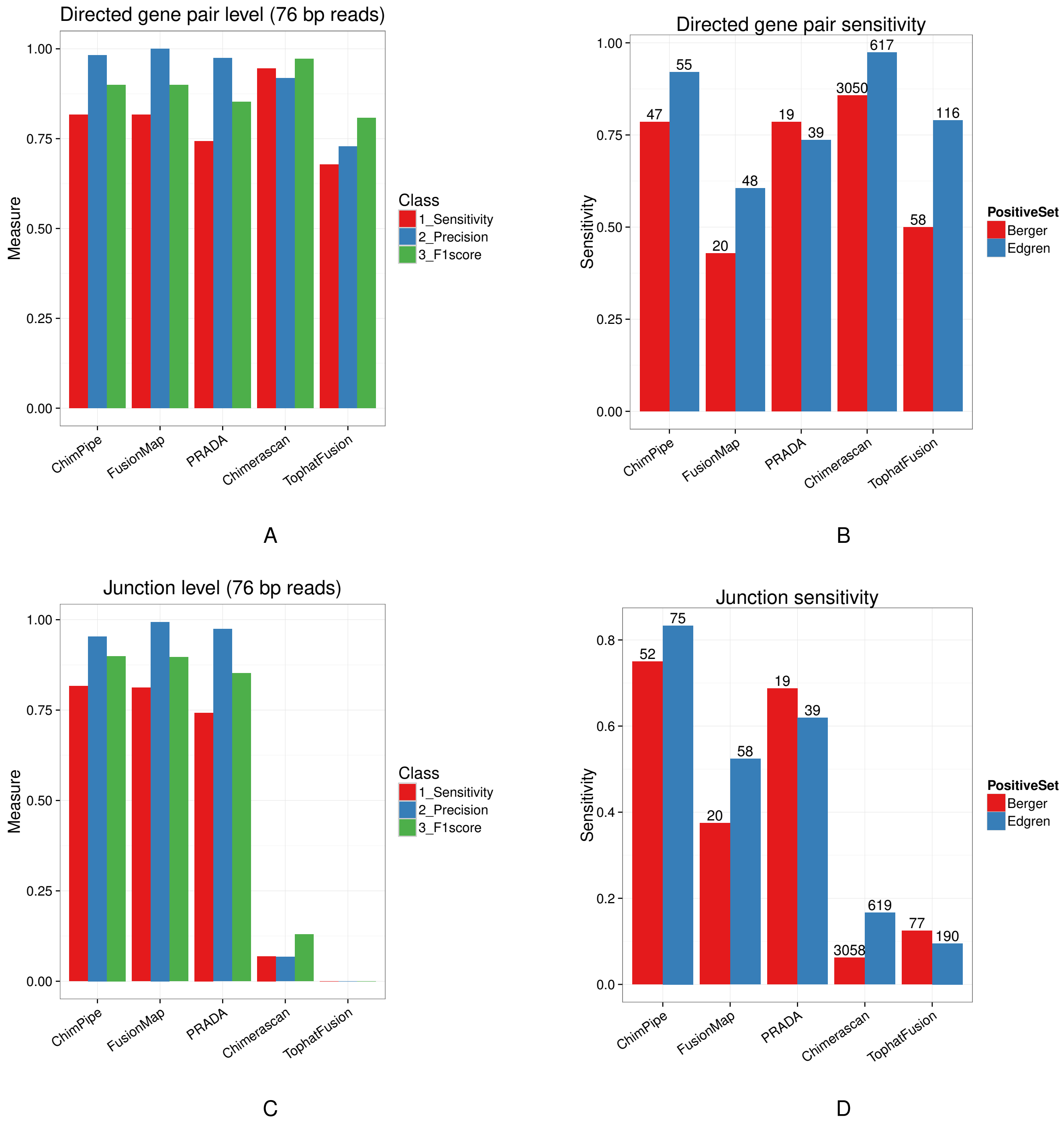

Figure S2. Benchmark results of 5 chimera detection programs on simulated data of different read lengths and on real data. On the top row **(A-D)** are the programs' performances at the gene pair level, and on the bottom row **(E-H)** at the junction level. The three first sets of barplots of each row are the results on simulated data, with sensitivity in first column, precision in second column and the F1score in third column, with different colors for 3 read lengths, while the last set of barplots of each row show the results on real data (Berger in red and Edgren in blue). According to simulated data, read length does not have a big impact on the results, except for Fusionmap which is better with 76bp reads. The sensitivity tends to decrease when the read length increases

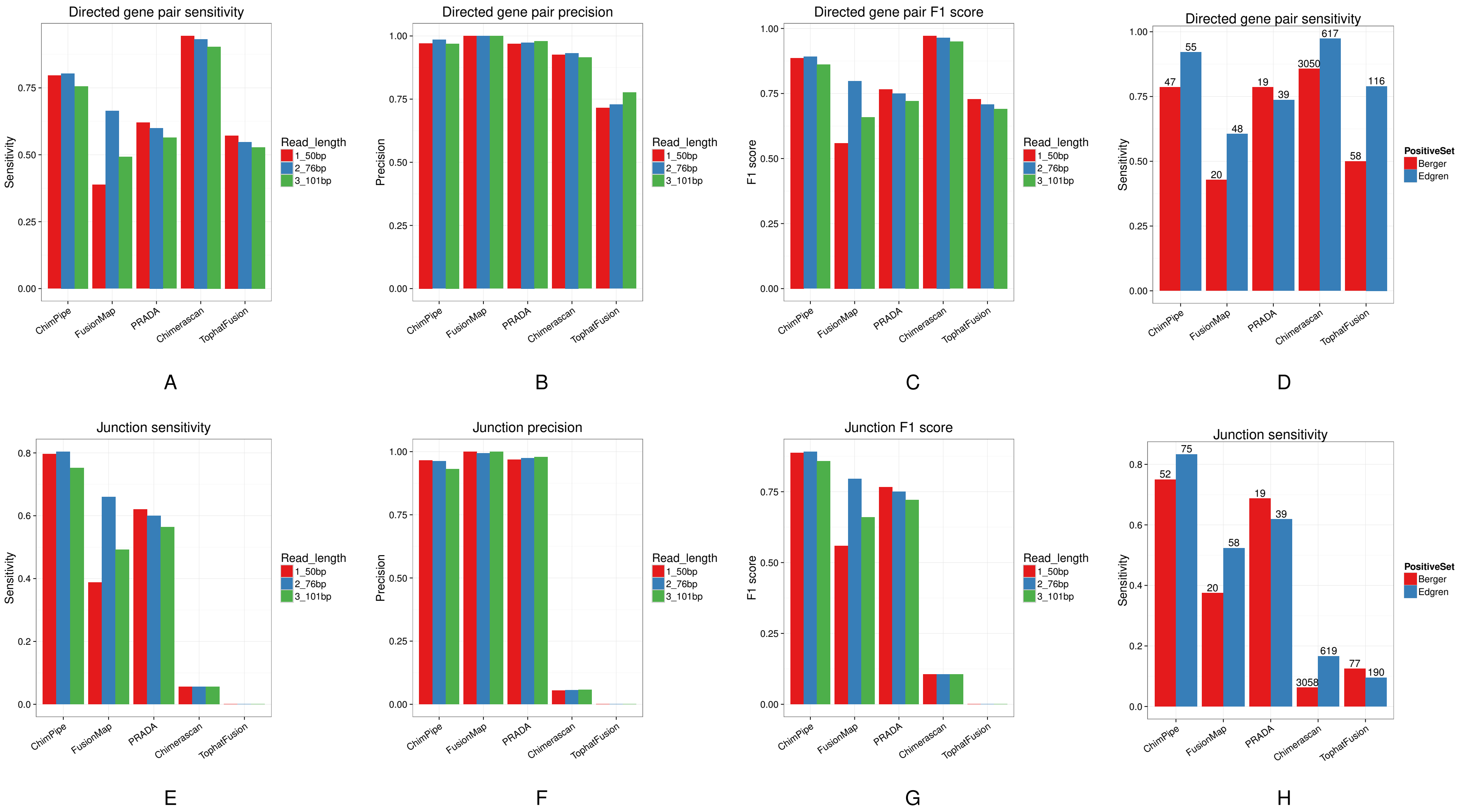

Figure S4. Distance between predicted and true junction on simulated data. This figure shows for the PE50 **(A)**, PE76 **(B)** and PE101 **(C)** simulated sets, and for each program, the distance between the predicted and the true/reference junction for predicted junctions belonging to TP gene pairs. The distance between the predicted and the true junction is computed as the sum of the distance between the predicted and the true donor/upstream/5' splitce sites and the distance between the predicted and the true acceptor/downstream/3' splice sites.

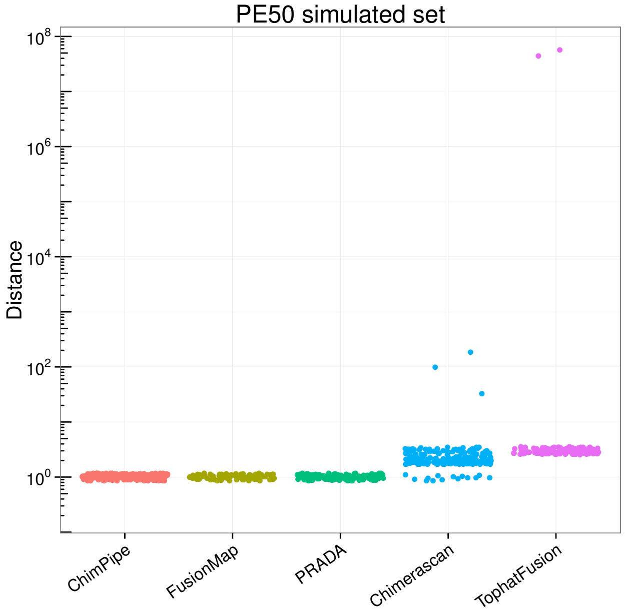

A

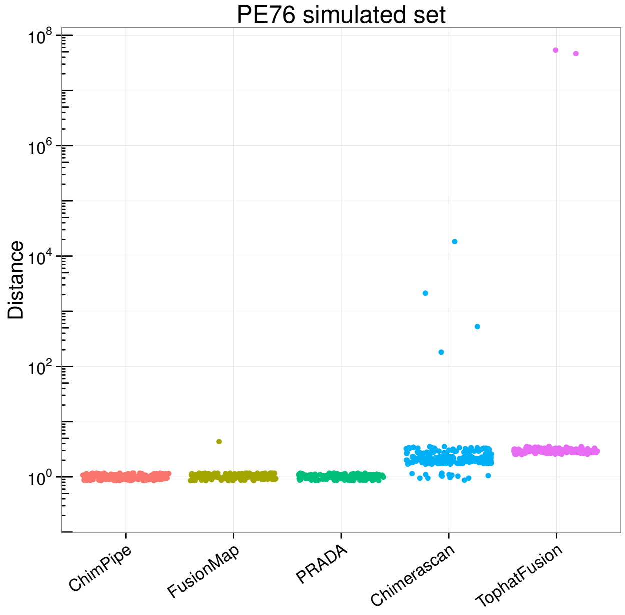

B

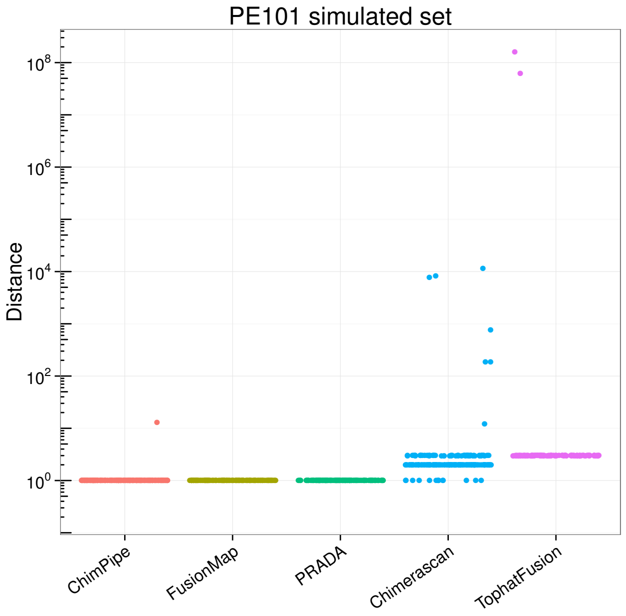

C

Figure S5. RT-PCR validation method. For each chimeric junction attempted to be validated by RT-PCR, 3 tests were actually performed, each of them requiring different pairs of primers: (1) the actual validation of the chimeric junction is done by doing RT-PCR on a cDNA library using a pair of primers that are located externally to the junction but in the exons overlapped by each part of the junction; (2) genomic DNA tests (starting from genomic DNA) are done for each of the parent gene and for the chimeric junction, in order to check whether a genomic rearrangement could explain the chimeric junction and to see if the parent genes are present at the DNA level; (3) mRNA checks are done for each parent gene (starting from cDNA and using pairs of primers specific to each gene) in order to check whether they are present at the mRNA level.

A) With internal exons

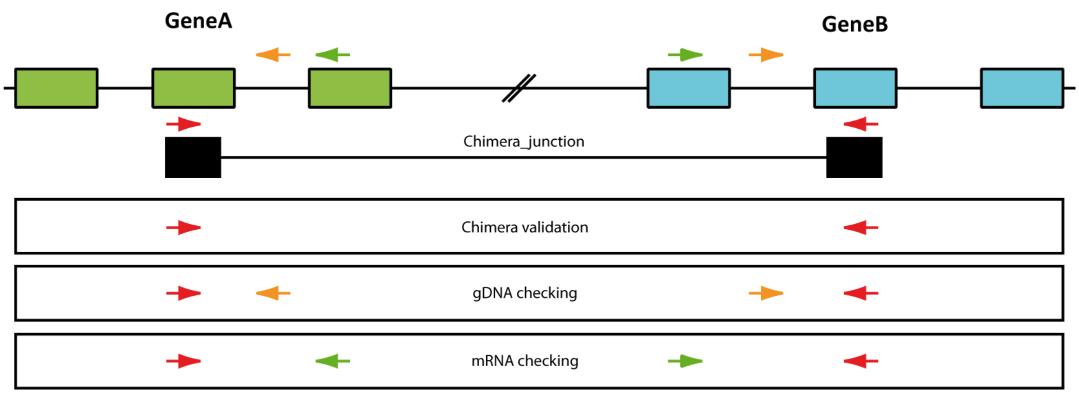

B) Without internal exons

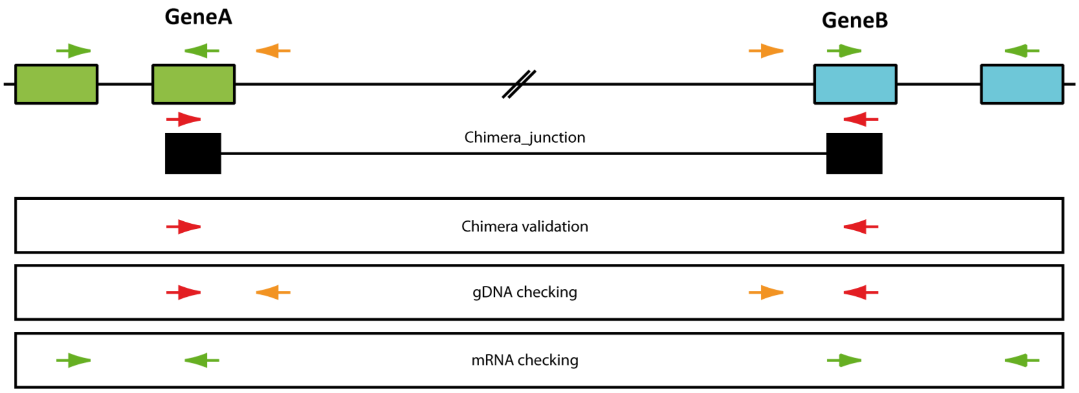

Figure S6. RT-PCR validation results for 4 chimeras in four cell lines (HeLa, HL60, MCF-7, K562). Here we show the products of the RT-PCR amplification of 4 chimeras and their parent genes, from the cDNAs of 4 different cell lines: HeLa (H), HL60 (6), MCF-7 (M) and K562 (K). For each chimeric junction we also provide a negative control (-) for comparison, and higlight the bands that show the presence of the chimeras and of the parent genes. For the 3 successfully validated cases (3 first ones, i.e. UBA2-WTIP, PICALM-SYTL2 and RPL38-TTYH2), the bands show the presence of the chimeric RNAs at the expected size, except for PICALM-SYTL2, which chimera size is higher than expected, and of all parent mRNAs except SYTL2, although this could be due to a low expression level of this gene.

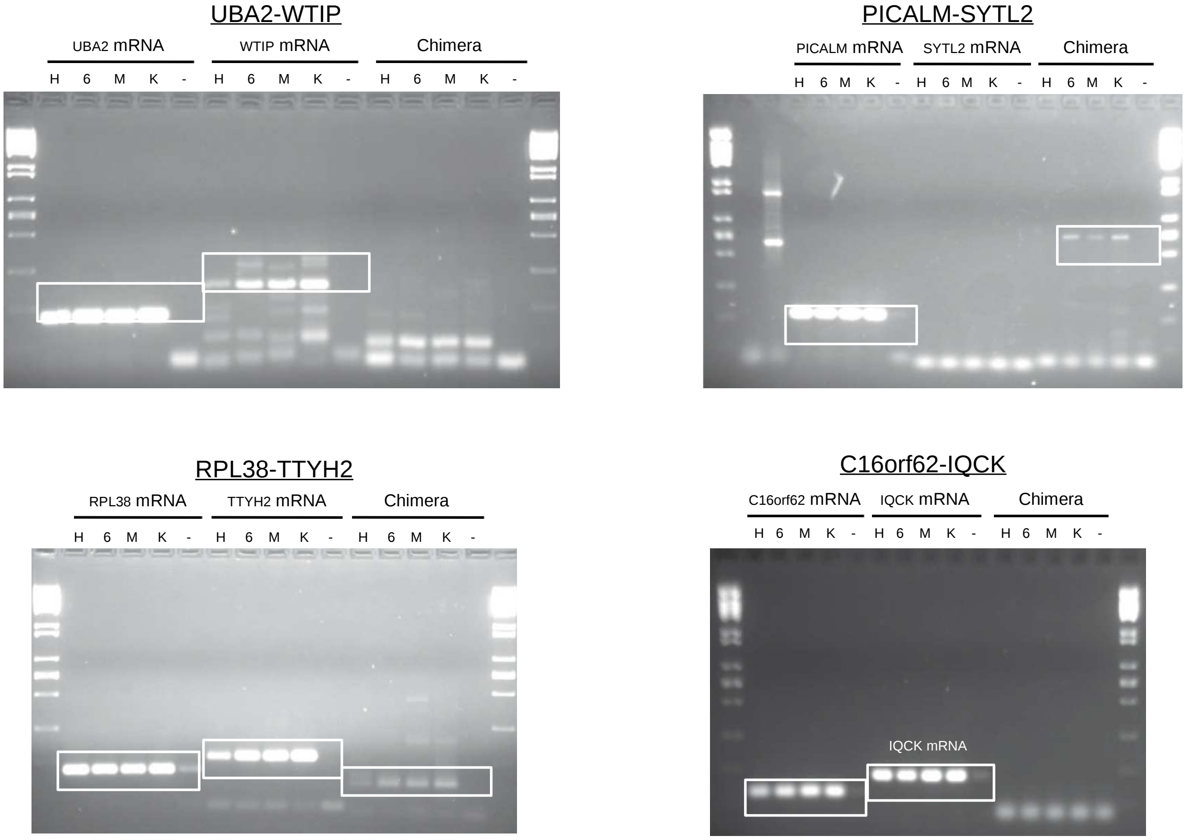

Figure S7. Genomic DNA analysis for the 3 successfully validated chimeras. For the 3 chimeras that were successfully validated by RT-PCR, we show the products of the genomic amplification of the 6 chimeras and their parent genes in the same 4 cell lines as the ones where the mRNA analysis was done (see supplementary Figure S5 above). These tests show that the parent genes are present at the DNA level, but not the chimeras. Indeed we see some unspecific amplification in the genomic DNA for the chimeras, but the band intensities are too low to consider them as genomic rearrangements. For the RPL38-TTYH2 chimera, there are some clearer unspecific products, but they are probably due to a primer contamination since they are also present in the negative control. H: HeLa, 6: HL60, M: MCF-7, K: K562, -: negative control.

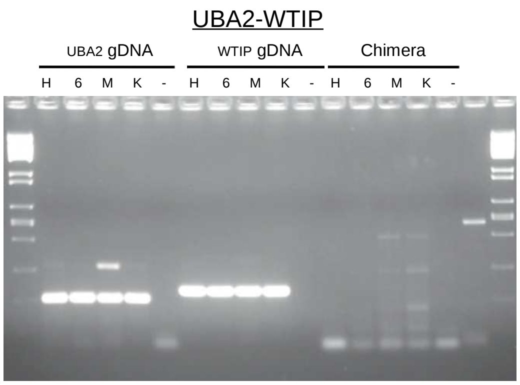

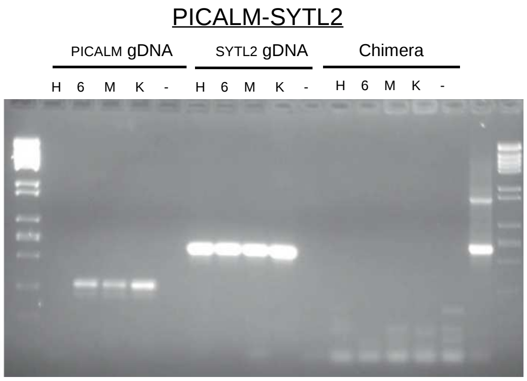

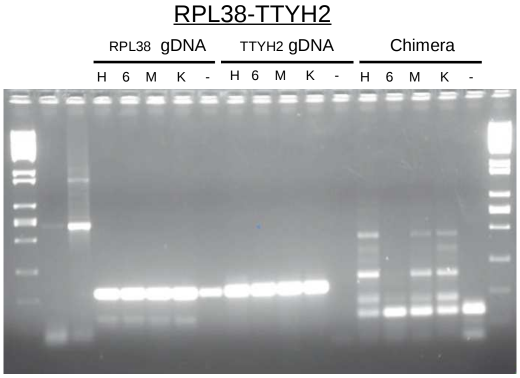

Figure S8. Colony PCR check and selection for RT-PCR validated chimeras. For the 3 RT-PCR validated chimeras, the RT-PCR products were purified from the gel bands, and cloned into pGEMTeasy vectors. E. coli bacterias were then transformed with these vectors, white colonies were selected and colony PCR was performed with the results indicated on the picture. Selected colonies (indicated by red boxes) were grown, plasmid purified and sent for sequencing.

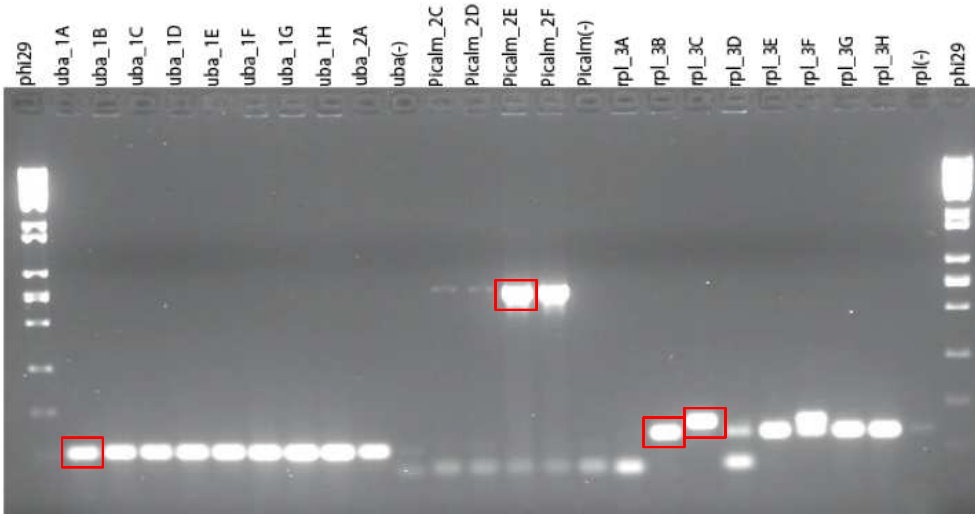

ChimPipe: Accurate detection of fusion genes and transcription-induced chimeras from RNA-seq data

Supplementary methods

[Making chimeric transcripts from the gene annotation](#)

[Simulating reads from transcripts](#)

[Obtaining chimeric junction coordinates from fusion genes](#)

[Running ChimPipe and state-of-the art programs](#)

[Benchmarking the programs](#)

[RT-PCR validation of ENCODE chimeras](#)

[Common recurrent chimeras between human and mouse](#)

For the programs' benchmarking we used the hg19 human genome available at the UCSC as our reference genome, and the Gencode v19 gene annotation from which we removed all small RNA genes (Additional file 2), as our reference transcriptome/annotation.

Making chimeric transcripts from the gene annotation

To make chimeric transcripts from the gencode v19 long gene annotation, we used ChimSim, that we developed and have made available here: https://github.com/Chimera-tools/ChimSim.

This program takes as input:

- an annotation file in gtf format

- a gem genome index file in gem format
- 5 numbers corresponding to the wanted numbers of read-through, intrachromosomal, inverted, interstrand and interchromosomal chimeric transcripts
- an optional list of biotypes to consider for the parent genes of the chimeras. It then outputs the wanted number of chimeric transcripts from the 5 classes, by only considering spliced genes of the wanted biotypes from the annotation.

This program then outputs the wanted number of chimeric transcripts from the 5 classes by sampling the wanted number of gene pairs for each class, then sampling a given transcript for each gene, and finally a given splice site for each transcript (a donor if the transcript is the first in the pair, an acceptor otherwise). For each (donor, acceptor) pair it then constructs the associated chimeric transcript by concatenating the 5' exons of the first (5') transcript until the chosen donor, and the 3' exons of the second (3') transcript from the chosen acceptor.

<u>Here is the command line that we used:</u>

```
ChimSim.sh gencode.v19.annotation.long.gtf \
Homo_sapiens.GRCh37.chromosomes.chr.M.gem 50 50 50 50 50 \
wanted_gnbt.txt
```

<u>where:</u>

- `wanted_gnbt.txt` is a 1 column file including the 'protein_coding' gene biotype

This resulted in a fasta file containing 250 chimeric transcripts, and whose header contained information about the 5' and 3' gene and transcript ids, the global and local coordinates of the chimeric junction and the chimera class.

Simulating reads from transcripts

Learning sequencing quality profile from Illumina PE data of the same read length

In order to produce simulated reads that are as close as possible to real Illumina reads, we generated read quality profiles from real data of the same read length using the `art_profiler_illumina` program of the ART suite (version 2.3.7), and then gave it as input to the `art_illumina` program of the same suite (see below). In order not to learn sample specific quality profiles and since `art_profiler_illumina` is able to average profiles learnt from several samples, we used 4 samples for each read length (8 fastq files in total since the data is PE): the 4 Edgren PE50 samples, 4 ENCODE PE76 samples and 2 ENCODE PE101 samples (GEO accession numbers provided in Additional file 2).

<u>Here is the command line that we used for a given read length:</u>

```
art_profiler_illumina illumina${rl}bp $readDir fastq.gz
```

where:

- `$rl` is the read length
- `$readDir` is a directory containing the fastq files of the 4 samples of read length $rl of which we want to learn the read quality profile

This resulted in 2 profile matrices for each read length, one for each mate, which are used by `art_illumina` for read simulation (see below).

Simulating Illumina unstranded PE reads

We used the `art_illumina` program of the ART suite version 2.3.7 to simulate Illumina non directional paired end reads from the 102,399 chimeric and non chimeric transcripts previously generated. Since we wanted to see the influence of the read length of the programs' behaviour, we used `art_illumina` three times, asking for PE50, PE76 and PE101 reads. We asked for a coverage of 20 in all cases (-f option, see below).

Here is the command line that we used for a given read length:

```
art_illumina -i $fasta --qprof1 $prof1 --qprof2 $prof2 -l $rl -f 20 \
-p -m $ism -s $iss -o chim_tr_seq_read_simulation
```

where:

- `$fasta` is the fasta file of the union of chimeric and non chimeric transcripts
- `$prof1` and `$prof2` are the read1 and read2 sequencing quality profiles generated by `art_profiler_illumina` program (see above)
- `$rl` is the read length (50, 76 or 101 depending on the set)
- `$ism` and `$iss` are respectively the mean and standard deviation of the insert size (200 and 20 for PE50, 250 and 25 for PE76, 300 and 30 for PE101).

With these parameters `art_illumina` generated two fastq files for each read length, one for each mate, with the following number of reads in each:

- 32,320,070 for 50bp reads,
- 21,092,450 for 76bp reads
- 15,730,360 for 101bp reads

Obtaining chimeric junction coordinates from fusion genes

In order to assess the ability of the chimera detection programs to detect exact chimeric junctions, we defined a protocol to identify the chimeric junction coordinates from RT-PCR validated fusion transcripts, such as the ones from the Edgren and the Berger cancer studies (see main text). We first downloaded the chimeric junction cDNA sequences from the supplementary material of their respective publications: Additional file 8 from the Edgren et al. study, and Additional file 1: Figures S4 and S5 from the Berger et al study. Since the cDNA sequences were not provided by the Kangaspeska et al study, we requested them from the authors. Then, we used the UCSC genome browser Blat tool to align the cDNAs to the hg19 human genome. For each cDNA, we identified the pair of non-overlapping partial alignments encompassing the complete cDNA sequence and connecting a donor and acceptor exon boundary from the two genes involved in the fusion gene (using as a reference the gencode v19 long gene annotation, see main text). The genomic positions of the donor and acceptor splice sites identified this way are considered to be the coordinates of the chimeric junction corresponding to the initial RT-PCR validated fusion transcript.
During this process, we discarded two validated fusion transcripts from the SK-BR-3 cell line: CSE1L-ENSG00000236127 and NFS1-PREX1. The first case was discarded since the gene ENSG00000236127 was not annotated in gencode v19 and the second one because it had a too short cDNA sequence on one side of the junction site, making this piece of sequence impossible to align with Blat.

Running ChimPipe and state-of-the art programs

Here we give the commands used for the 5 programs on the simulated datasets, but the parameters were the same for the real datasets.
<u>Chimpipe version 0.9.3 was run with this command, using SAMtools 1.2 and bedtools 2.21:</u>

```
ChimPipe.sh --fastq_1 $read1 --fastq_2 $read2 -g $genome -a $annot -t
$annot.junctions.gem -k $annot.junctions.keys --sample-id sim
--threads 4 --similarity-gene-pairs $gnsim --log debug --no-cleanup
```

where:

- `$read1` is the fastq.gz file of the first reads of the pairs
- `$read2` is the fastq.gz file of the second reads of the pairs
- $genome is the genome index file
- $annot is the annotation file
- `$annot.junctions.gem` is the annotated transcriptome index file
- `$annot.junctions.keys` is the transcriptome to genome coordinate conversion key file
- `$gnsim` is the gene sequence similarity file

FusionMap version 8.0.2.32 was run with this command, using mono version 2.10.9:

```
mono FusionMap.exe --semap $baseDir hg19 gencode19 $config
```

where:

- `$baseDir` is the directory containing the indices of the genome and transcriptome
- `$config` is a configuration file containing the following options:
  PairedEnd=True
  RnaMode=True
  ThreadNumber=4
  FileFormat=FASTQ
  CompressionMethod=Gzip
  Gzip=True
  MinimalFusionSpan=2000
  OutputFusionReads=True

and the output file considered for the evaluation was `01_chimtr_$rl\_InputFastq_gencode19.FusionReport.txt` for each read length `$rl`.

For PRADA version 1.2 we ran the following 3 successive steps, using GCC 4.7.2, Perl 5.16.3, Java/1.7.0_10, Python 2.7.3 and pysam 0.8.0:

1. Pre-processing step to make mapping script:
   ```
   prada-preprocess-bi -conf $config -inputdir $inputdir -sample
   chimtr -tag chimtr -platform illumina -intermediate yes -step
   2_e1_1 -submit no
   ```
2. Run mapping script step:
   ```
   Bash prada_prep_2015_Dec_18.1450430506.42.pbs
   ```
3. Compute fusion step:

```
prada-fusion -bam chimtr.withRG.GATKRecalibrated.flagged.bam
-conf $config -tag chimtr -junL $pcent -outdir $outdir
```

where:

- `$config` is the configuration file containing reference files used by PRADA such as reference genome, annotation, SNP set, ...etc. Since those are 13 files and we found no script or document explaining how to obtain them for any annotation, we kept the ensembl64 files provided by PRADA
- `$inputdir` is the input directory containing the fastq files
- `chimtr.withRG.GATKRecalibrated.flagged.bam` is the bam file output by the previous step
- `$pcent` is 80% of the read length as recommended
- `$outdir` is the output directory

and the output file considered for the evaluation was `chimtr.fus.candidates.txt`

Chimerascan version 0.4.5 was run with this command, using Bowtie 0.12.7 and the python2.7 libraries provided by Chimerascan:

```
python chimerascan_run.py -p 4 --isize-mean=$ism --isize-stdev=$iss
-v --quals solexa $index $read1 $read2 $PWD
```

where:

- `$ism` is the mean insert size
- `$iss` is the insert size standard deviation
- `$index` is the index file of the genome and annotation
- `$read1` is the fastq.gz file of the first reads of the pairs
- `$read2` is the fastq.gz file of the second reads of the pairs

and the output file considered for the evaluation was `chimeras.bedpe`.

For TopHatFusion version 2.0.12 we ran the following 2 successive steps, the first one for each read length separately, and the second one for all read lengths together, using Bowtie 1.1.0, TopHat 2.1.0 and Blast 2.2.29, and having previously downloaded the blast database, as recommended by the authors:

1. Mapping step to find all the possible fusions :

```
tophat -o tophat_sim -p 4 --fusion-search --bowtie1
--no-coverage-search -r $mid --mate-std-dev $iss
--fusion-min-dist 100000 --max-intron-length 100000
--fusion-anchor-length 10 --fusion-ignore-chromosomes chrM
$index $read1 $read2
```

2. Filtering step to discard false positives
   `tophat-fusion-post -p 4 --num-fusion-reads 1 --num-fusion-pairs 2 --num-fusion-both 5 $index`

where:

- `$mid` is the mate inner distance, which is the insert length minus twice the read length
- `$iss` is the standard deviation of the above distance, which we took as the insert length standard deviation
- `$index` is the indexed genome file
- `$read1` is fastq.gz file of the first reads of the pairs
- `$read2` is the fastq.gz file of the second reads of the pairs

and the output file considered for the evaluation was `tophatfusion_out/result.txt`
In order to evaluate the programs in an automatic way, their output was first made into chimpipe format, and the same benchmark script was applied to each of them, taking as input the reference sets of junctions and the gencode v19 long annotation (see below).

Benchmarking the programs

To benchmark the programs' predicted chimeric junctions on both simulated and real data, we used the ChimBench program that we developed and made accessible here: https://github.com/Chimera-tools/ChimBench.
This program assesses chimeric junctions where the two parts strandly overlap exons, and takes as input three flat files:

- a tsv file with header with the reference chimeric junctions in the 1st column, in chimpipe format (donchrom_donpos_donstrand:accchrom_accpos_accstrand)
- a tsv file with header with the predicted chimeric junctions in the 1st column, in chimpipe format
- A gtf or gff version 2 file of the annotated exons with gene_id and transcript_id as the first two keys in the 9th field

It then produces:

- on the standard error a tabulated report with the number of junctions and gene pairs in each set, the number of junctions and gene pairs in common, the number of junctions and gene pairs that are in one and not in the other set, and a sensitivity and a precision measure at both the gene pair and the junction levels (although for positive sets this

precision is an underestimate of the true one since we do not know whether there are other chimeras to be found in this set)

- a 1 column file called `common.txt` with the coordinates of the common chimeric junctions
- an 8 column tsv file called `ref_junc_belonging_to_common_gnpairs_vs_pred_same.tsv` with the predicted junctions (same as 2nd input file) but with information about all the reference junctions sharing the same chromosome and strand for the two parts of the junction, their donor distance to the predicted junction donor, their acceptor distance to the predicted junction acceptor, the sum of those and the subset of sums that are minimum together with their associated reference junctions
- other intermediate gff and tsv files

RT-PCR validation of ENCODE chimeras

ChimPipe 0.9.3 was run on the 106 ENCODE CSHL PE RNA-seq experiments (Additional file 1: Table S7) with default parameters and the --no-clean-up option. We then used the candidate chimera file from each experiment to select the chimeras that did not have a strong exonic sequence similarity between the 2 connected genes (a high sequence similarity being defined as a blast hit of more than 30 bp with more than 80% identity) and with the support of at least 10 staggered split-reads and 5 discordant PE reads. We then gathered all such chimeras across the 106 experiments.

Total DNA and RNA were extracted independently from four different cell types (HeLa, HL60, K562 and MCF7) using DNeasy® Blood & Tissue kit (QIAGEN) and SV Total RNA Isolation System (Promega), respectively. Then, 1 µg of total RNA was used to synthetize cDNA using ImProm-II™ Reverse Transcription System (Promega) according to manufacturer's instructions.

Several pair of primers were designed to validate the chimeric junctions. A first pair of primers was designed to analyze chimeric junctions in the cDNA preparations, and to discard a genomic reorganization using genomic DNA (gDNA) as template. Another pair of primers was then used to confirm the presence of each gene individually in gDNA and the presence of each individual parental transcripts in the cDNA preparations (Additional file 1: Figure S5). PCRs were performed using GoTaq Green Master Mix (Promega). The PCR products from the three

positive chimeric junctions were gel purified using Wizard® SV Gel and PCR Clean-Up System (Promega) and cloned into pGEMT-easy (Promega), Finally, cloned chimeric junctions were sequenced at the Genomic facility of the Instituto de Investigación Biomédicas Alberto Sols.

For the most interesting case, the UBA-WTIP chimera, primers were designed in order to identify each possible complete chimeric transcript (Q1, Q3 and Q5 in Figure 6), based on the annotation of both parent genes and the exact localization of the chimeric junction. PCRs and sequencing were performed as described above.

Additional file 1: Tables S8-9 provide the primers' sequences and their characteristics.

Common recurrent chimeras between human and mouse

ChimPipe 0.9.2 was run with default parameters (--no-clean-up option) on each human and mouse ENCODE CSHL PE RNA-seq experiment (bio-replicate of a sample) requiring each reported junction to be supported by at least 1 split-read and 1 discordant PE read (starting from the candidate chimera file output by ChimPipe). For human we used the hg19 genome assembly and the gencode v19 long gene annotation, and for mouse we used the mm9 genome assembly and the ensembl v65 long gene annotation.

A chimera was defined as detected in a given sample if the sum of its number of supporting split-reads in the 2 bio-replicates of the sample was at larger or equal to 2. A recurrent chimera was defined as a chimera detected in at least 2 samples.
