# Supplementary material for: ChimPipe: accurate detection of fusion genes and transcription-induced chimeras from RNA-seq data
Source: BMC Genomics. 2017 Jan 3;18:7. doi: 10.1186/s12864-016-3404-9 (PMC5209911; doi:10.1186/s12864-016-3404-9)
Supplement: Additional file 4 — Junction sequences for 3 RT-PCR validated chimeras (DOCX 4.39 kb) [file 12864_2016_3404_MOESM4_ESM.docx]

>Uba-Wtip

CAATGGCAGTGATGATGGAGCTCAGCCCTCCACCTCCACAGGCATTTGCATCAAGTGTGGGCTTGGCATCTAC

>Picalm-Sytl

TCCTGTAATGACGCAACCAACCTTAATATACAGCCAGCCTGTCATGAGACCTCCAAACCCCTTTGGCCCTGTATCAGGAGCACAGGTAGGTGATGCTCTGATGCTGCTGCCTCTTATGAATTGACGACAGGTGCAGAGAGAGAATAAACTGTGTCTAAGACTGGACCAGAGAGTGAGTGTGCTGGAAGCCACCATAGGAACAAGCGACCTGTTTGGCAAGGAATACAGCATCCTTCTGAGAGCCTCTTCTCGACACTATCCCACAGACCAGGACTTCCTCATGGGAAAGAGCACTTAGGATCTTGCTATCAGCTGCCCTAAACTTTTGCTGAGCCAAAATAAGACTTTGATGCAGAACTGAACAAGATATTTCATTTTTGTGTGTTTTAATCTTCCTGGTTGCAGTCTTAGTTGTTCAGCCCTGAGAATTTTGAGCCACATTTGTTGCTATTATTTTTGCATGCACTTTTCAAAATGATTGACTTAAGCTTCCTGACTGAAGAGGAACAAGAGGCCATCATGAAGGTTTTGCAGCGGGATGCTGCTCTG

>Rpl38-Ttyh2

AAGGACTTCCTGCTCACAGCCCGACGAAAGGATGCCAAATTCGCTGCTGTTCCTGGGGCTGGTGGCCGCCGTCTGCCTGGGCCTGAACCTCATCTTCCTTG
