# Supplementary material for: ChimPipe: accurate detection of fusion genes and transcription-induced chimeras from RNA-seq data
Source: BMC Genomics. 2017 Jan 3;18:7. doi: 10.1186/s12864-016-3404-9 (PMC5209911; doi:10.1186/s12864-016-3404-9)
Supplement: Additional file 5 — UBA2-WTIP transcript isoform sequences (DOCX 142kb) [file 12864_2016_3404_MOESM5_ESM.docx]

>HeLa_LE2_5 (Q1)

GGTTTCTCCCACATCGACCTGATTGATCTGGATACTATTGATGTAAGCAACCTCAACAGACAGTTTTTGTTTCAATAGAAACATGTTGGAAGATCAAAGGCACAGGTTGCCAAGGAAAGTGTACTGCAGTTTTACCCGAAAGCTAATATCGTTGCCTACCATGACAGCATCATGAACCCTGACTATAATGTGGAATTTTTCCGACAGTTTATACTGGTTATGAATGCTTTAGATAACAGAGCTGCCCGAAACCATGTTAATAGAATGTGCCTGGCAGCTGATGTTCCTCTTATTGAAAGTGGAACAGCTGGGTATCTTGGACAAGTAACTACTATCAAAAAGGGTGTGACCGAGTGTTATGAGTGTCATCCTAAGCCGACCCAGAGAACCTTTCCTGGCTGTACAATTCGTAACACACCTTCAGAACCTATACATTGCATCGTTTGGGCAAAGTACTTGTTCAACCAGTTGTTTGGGGAAGAAGATGCTGATCAAGAAGTATCTCCTGACAGAGCTGACCCTGAAGCTGCCTGGGAACCAACGGAAGCCGAAGCCAGAGCTAGAGCATCTAATGAAGATGGTGACATTAAACGTATTTCTACTAAGGAATGGGCTAAATCAACTGGATATGATCCAGTTAAACTTTTTACCAAGCTTTTTAGAGATGACATCAGGTATCTGTTGACAATGGACAAACTATGGCGGAAAAGGAAACCTCCAGTTCCGTTGGACTGGGCTGAAGTACAAAGTCAAGGAGAAGAAACGAATGCATCAGATCAACAGAATGAACCCCAGTTAGGCCTGAAAGACCAGCAGGTTCTAGATGTAAAGAGCTATGCACGTCTTTTTTCAAAGAGCATCGAGACTTTGAGAGTTCATTTAGCAGAAAAGGGGGATGGAGCTGAGCTCATATGGGATAAGGATGACCCATCTGCAATGGATTTTGTCACCTCTGCTGCAAACCTCAGGATGCATATTTTCAGTATGAATATGAAGAGTAGATTTGATATCAAATCAATGGCAGGGAACATTATTCCTGCTATTGCTACTACTAATGCAGTAATTGCTGGGTTGATAGTATTGGAAGGATTGAAGATTTTATCAGGAAAAATAGACCAGTGCAGAACAATTTTTTTGAATAAACAACCAAACCCAAGAAAGAAGCTTCTTGTGCCTTGTGCACTGGATCCTCCCAACCCCAATTGTTATGTATGTGCCAGCAAGCCAGAGGTGACTGTGCGGCTGAATGTCCATAAAGTGACTGTTCTCACCTTACAAGACAAGATAGTGAAAGAAAAATTTGCTATGGTAGCACCAGATGTCCAAATTGAAGATGGGAAAGGAACAATCCTAATATCTTCCGAAGAGGGAGAGACGGAAGCTAATAATCACAAGAAGTTGTCAGAATTTGGAATTAGAAATGGCAGCCGGCTTCAAGCAGATGACTTCCTCCAGGACTATACTTTATTGATCAACATCCTTCATAGTGAAGACCTAGGAAAGGACGTTGAATTTGAAGTTGTTGGTGATGCCCCGGAAAAGGTGGGGCCCAAACAAGCTGAAGATGCTGCCAAAAGCATAACCAATGGCAGTGATGGTGGAGCTCAGCCCTCCACCTCCACAGGCATTTGCATCAAGTGTGGGCTTGGCATCTACGGAGCCCAGCAGGCGTGCCAGGCAATGGGGAGTCTTTATCACACTGACTGCTTCACCTGCGACTCGTGTGGGAGACGACTCCGTGGGAAGGCGTTCTACAACGTGGGTGAGAAAGTGTACTGCCAGGAGGACTTCCTGTACTCCGGGTTCCAGCAGACGGCCGACAAATGCAGCGTGTGTGGACATCTCATCATGGAAATGATCCTGCAGGCCCTGGGCAAGTCCTACCACCCAGGCTGCTTCCGGTGCTCCGTGTGCAATGAGTGCCTGGACGGGGTTCCCTTCACCGTGGACGTGGAGAACAACATCTACTGCGTGCGAGACTATCACACGGTTTTTGCACCAAAATGCGCCTCCTGTGCCCGTCCTATCCTCCCTGCACAGGGCTGCGAGACAACCATCCGTGTGGTGTCCATGGACAGAGACTACCACGTGGCATGTTACCACTGTGAGGACTGCGGGCTGCAGCTGAGCGGGGAGGAGGGACGCCGTTGCTATCCCCTGGCGGGCCACCTACTGTGTCGTCGTTGCCACCTGCGGCGCCTCCAACCTGGGCCTCTTCCCTCACCCACTGTGCACGTCACTGAGCTCTGAGCA

>HL60_LE2_4 (Q1)

GGTTTCTCCCACATCGACCTGATTGATCTGGATACTATTGATGTAAGCAACCTCAACAGACAGTTTTTGTTTCAAAAGAAACATGTTGGAAGATCAAAGGCACAGGTTGCCAAGGAAAGTGTACTGCAGTTTTACCCGAAAGCTAATATCGTTGCCTACCATGACAGCACCATGAACCCTGACTATAATGTGGAATTTTTCCGACAGTTTATACTGGTTATGAATGCTTTAGATAACAGAGCTGCCCGAAACCATGTTAATAGAATGTGCCTGGCAGCTGATGTTCCTCTTATTGAAAGTGGAACAGCTGGGTATCTTGGACAAGTAACTACTATCAAAAAGGGTGTGACCGAGTGTTATGAGTGTCATCCTAAGCCGACCCAGAGAACCTTTCCTGGCTGTACAATTCGTAACACACCTTCAGAACCTATACATTGCATCGTTTGGGCAAAGTACTTGTTCAACCAGTTGTTTGGGGAAGAAGATGCTGATCAAGAAGTATCTCCTGACAGAGCTGACCCTGAAGCTGCCTGGGAACCAACGGAAGCCGAAACCAGAGCTAGAGCATCTAATGAAGATGGTGACATTAAACGTATTTCTACTAAGGAATGGGCTAAATCAACTGGATATGATCCAGTTAAACTTTTTACCAAGCTTTTTAAAGATGACATCAGGTATCTGTTGACAATGGACAAACTATGGCGGAAAAGGAAACCTCCAGTTCCGTTGGACTGGGCTGAAGTACAAAGTCAAGGAGAAGAAACGAATGCATCAGATCAACAGAATGAACCCCAGTTAGGCCTGAAAGACCAGCAGGTTCTAGATGTAAGGAGCTATGCACGTCTTTTTTCAAAGAGCATCGAGACTTTGAGAGTTCATTTAGCAGAAAAGGGGGATGGAGCTGAGCTCATATGGGATAAGGATGACCCATCTGCAATGGATTTTGTCACCTCTGCTGCAAACCTCAGGATGCATATTTTCAGTATGAATATGAAGAGTAGATTTGATATCAAATCAATAGCAGGGAACATTATTCCTGCTATTGCTACTACTAATGCAGTAATTGCTGGGTTGATAGTATTGGAAGGATTGAAGATTTTATCAGGAAAAATAGACCAGTGCAGAACAATTTTTTTGAATAAACAACCAAACCCAAGAAAGAAGCTTCTTGTGCCTTGTGCACTGGATCCTCCCAACCCCAATTGTTATGTATGTGCCAGCAAGCCAGAGGTGACTGTGCGGCTGAATGTCCATAAAGTGACTGTTCTCACCTTACAAGACAAGATAGTGAAAGAAAAATTTGCTATGGTAGCACCAGATGTCCAAATTGAAGATGGGAAAGGAACAATCCTAATATCTTCCGAAGAGGGAGAGACGGAAGCTAATAACCACAAGAAGTTGTCAGAATTTGGAATTAGAAATGGCAGCCGGCTTCAGGCAGATGACTTCCTCCAGGACTATACTTTATTGATCAACATCCTTCATAGTGAAGACCTAGGAAAGGACGTTGAATTTGAAGTTGTTGGTGATGCCCCGGAAAAAGTGGGGCCCAAACAAGCTGAAGATGCTGCCAAAAGCATAACCAATGGCAGTGATGATGGAGCTCAGCCCTCCACCTCCACAGGCATTTGCATCAAGTGTGGGCTTGGCATCTACGGAGCCCAGCAGGCGTGCCAGGCAATGGGGAGTCTTTATCACACTGACTGCTTCACCTGCGACTCGTGTGGGAGACGACTCCGTGGGAAGGCGTTCTACAACGTGGGTGAGAAAGTGTACTGCCAGGAGGACTTCCTGTACTCCGGGTTCCAGCAGACGGCCGACAAATGCAGCGTGTGTGGACATCTCATCATGGAAATGATCCTGCAGGCCCTGGGCAAGTCCTACCACCCAGGCTGCTTCCGGTGCTCCGTGTGCAATGAGTGCCTGGACGGGGTTCCCTTCACCGTGGACGTGGAGAACAACATCTACTGCGTGCGAGACTATCACACGGTTTTTGCACCAAAGTGCGCCTCCTGTGCCCGTCCTATCCTCCCTGCACAGGGCTGCGAGACAACCATCCGTGTGGTGTCCATGGACAGAGACTACCACGTGGCATGTTACCACTGTGAGGACTGCGGGCTGCAGCTGAGCGGGGAGGAGGGACGCCGTTGCTATCCCCTGGCGGGCCACCTACTGTGTCGTCGTTGCCACCTGCGGCGCCTCCAACCTGGGCCTCTTCCCTCACCCACTGTGCACGTCACTGAGCTCTGAGCA

>HL60_LE2_6 (Q3)

GGTTTCTCCCACATCGACCTGCTACCAGGGTCTCACTATTTTGCTTAGGCTGGTCTTGAAGGTCTGGGCTCAAGCGATTCTCTCACTTTGTCCTCCCAAAGTGTTGGGATTACAGATTGATCTGGATACTATTGATGTAAGCAACCTCAACAGACAGTTTTTGTTTCAAAAGAAACATGTTGGAAGATCAAAGGCACAGGTTGCCAAGGAAAGTGTACTGCAGTTTTACCCGAAAGCTAATATCGTCGCCTACCATGACAGCATCATGAACCCTGACTATAATGTGGAATTTTTCCGACAGTTTATACTGGTTATGAATGCTTTAGATAACAGAGCTGCCCGAAACCATGTTAATAGAATGTGCCTGGCAGCTGATGTTCCTCTTATTGAAAGTGGAACAGCTGGGTATCTTGGACAAGTAACTACTATCAAAAAGGGTGTGACCGAGTGTTATGAGTGTCATCCTAAGCCGACCCAGAGAACCTTTCCTGGCTGTACAATTCGTAACACACCTTCAGAACCTATACATTGCATCGTTTGGGCAAAGTACTTGTTCAACCAGTTGTTTGGGGAAGAAGATGCTGATCAAGAAGTATCTCCTGACAGAGCTGACCCTGAAGCTGCCTGGGAACCAACGGAAGCCGAAGCCAGAGCTAGAGCATCTAATGAAGATGGTGACATTAAACGTATTTCTACTAAGGAATGGGCTAAATCAACTGGATATGATCCAGTTAAACTTTTTACCAAGCTTTTTAAAGATGACATCAGGTATCTGTTGACAATGGACAAACTATGGCGGAAAAGGAAACCTCCAGTTCCGTTGGACTGGGCTGAAGTACAAAGTCAAGGAGAAGAAACGAATGCATCAGATCAACAGAATGAACCCCAGTTAGGCCTGAAAGACCAGCAGGTTCTAGATGTAAAGAGCTATGCACGTCTTTTTTCAAAGAGCATCGAGACTTTGAGAGTTCATTTAGCAGAAAAGGGGGATGGAGCTGAGCTCATATGGGATAAGGATGACCCATCTGCAATGGATTTTGTCACCTCTGCTGCAAACCTCAGGATGCATATTTTCAGTGTGAATATGAAGAGTAGATTTGATATCAAATCAATGGCAGGGAACATTATTCCTGCTATTGCTACTACTAATGCAGTAATTGCTGGGTTGATAGTATTGGAAGGATTGAAGATTTTATCAGGAAAAATAGACCAGTGCAGAACAATTTTTTTGAATAAACAACCAAACCCAAGAAAGAAGCTTCTTGTGCCTTGTGCACTGGATCCTCCCAACCCCAATTGTTATGTATGTGCCAGCAAGCCAGAGGTGACTGTGCGGCTGAATGTCCATAAAGTGACTGTTCTCACCTTACAAGACAAGATAGTGAAAGAAAAATTTGCTATGGTAGCACCAGATGTCCAAATTGAAGATGGGAAAGGAACAATCCTAATATCTTCCGAAGAGGGAGAGACGGAAGCTAATAATCACAAGAAGTTGTCAGAATTTGGAATTAGAAATGGCAGCCGGCTTCAAGCAGATGACTTCCTCCAGGACTATACTTTATTGATCAACATCCTTCATAGTGAAGACCTAGGAAAGGACGTTGAATTTGAAGTTGTTAGTGATGCCCCGGAAAAAGTGGGGCCCAAACAAGCTGAAGATGCTGCCAAAAGCATAACCAATGGCAGTGATGATGGAGCTCAGCCCTCCACCTCCACAGGCATTTGCATCAAGTGTGGGCTTGGCATCTACGGAGCCCAGCAGGCGTGCCAGGCAATGGGGAGTCTTTATCACACTGACTGCTTCACCTGCGACTCGTGTGGGAGACGACTCCGTGGGAAGGCGTTCTACAACGTGGGTGAGAAAGTGTACTGCCAGGAGGACTTCCTGTACTCCGGGTTCCAGCAGACGGCCGACAAATGCAGCGTGTGTGGACATCTCATCATGGAAATGATCCTGCAGGCCCTGGGCAAGTCCTACCACCCAGGCTGCTTCCGGTGCTCCGTGTGCAATGAGTGCCTGGACGGGGTTCCCTTCACCGTGGACGTGGAGAACAACATCTACTGCGTGCGAGACTATCACACGGTTTTTGCACCAAAATGCGCCTCCTGTGCCCGTCCTATCCTCCCTGCACAGGGCTGCGAGACAACCATCCGTGTGGTGTCCATGGACAGAGACTACCACGTGGCATGTTACCACTGTGAGGACTGCGGGCTGCAGCTGAGCGGGGAGGAGGGACGCCGTTGCTATCCCCTGGCGGGCCACCTACTGTGTCGTCGTTGCCACCTGCGGCGCCTCCAACCTGGGCCTCTTCCCTCACCCACTGTGCACGTCACTGAGCTCTGAGCA

>MCF7_5 (Q5)

TGCAGTTTTACCCGAAAGCTAATATCGTTGCCTACCATGACAGCATCATGAACCCTGACTATAATGTGGAATTTTTCCGACAGTTTATACTGGTTATGAATGCTTTAGATAACAGAGCTGCCCGAAACCATGTTAATAGAATGTGCCTGGCAGCTGATGTTTCTCTTATTGAAAGTGGAACAGCTGGGTATCTTGGACAAGTAACTACTATCAAAAAGGGTGTGACCGAGTGTTATGAGTGTCATCCTAAGCCGACCCAGAGAACCTTTCCTGGCTGTACAATTCGTAACACACCTTCGGAACCTATACATTGCATCGTTTGGGCAAAGTACTTGTTCAACCAGTTGTTTGGGGAAGAAGATGCTGATCAAGAAGTATCTCCTGACAGAGCTGACCCTGAAGCTGCCTGGGAACCAACGGAAGCCGAAGCCAGAGCTAGAGCATCTAATGAAGATGGTGACATTAAACGTATTTCTACTAAGGAATGGGCTAAATCAACTGGATATGATCCAGTTAAACTTTTTACCAAGCTTTTTAAAGATGACATCAGGTATCTGTTGACAATGGACAAACTATGGCGGAAAAGGAAACCTCCAGTTCCGTTGGACTGGGCTGAAGTACAAAGTCAAGGAGAAGAAACGAATGCATCAGATCAACAGAATGAACCCCAGTTAGGCCTGAAAGACCAGCAGGTTCTAGATGTAAAGAGCTATGCACGTCTTTTTTCAAAGAGCATCGAGACTTTGAGAGTTCATTTAGCAGAAAAGGGGGATGGAGCTGAGCTCATATGGGATAAGGATGACCCATCTGCAATGGATTTTGTCACCTCTGCTGCAAACCTCAGGATGCATATTTTCAGTATGAATATGAAGAGTAGATTTGATATCAAATCAATGGCAGGGAACATTATTCCTGCTATTGCTACTACTAATGCAGTAATTGCTGGGTTGATAGTATTGGAAGGATTGAAGATTTTATCAGGAAAAATAGACCAGTGCAGAACAATTTTTTTGAATAAACAACCAAACCCAAGAAAGAAGCTTCTTGTGCCTTGTGCACTGGATCCTCCCAACCCCAATTGTTATGTATGTGCCAGCAAGCCAGAGGTGACTGTGCGGCTGAATGTCCATAAAGTGACTGTTCTCACCTTACAAGACAAGATAGTGAAAGAAAAATTTGCTATGGTAGCACCAGATGTCCAAATTGAAGATGGGAAAGGAACAATCCTAATATCTTCCGAAGAGGGAGAGACGGAAGCTAATAATCACAAGAAGTTGTCAGAATTTGGAATTAGAAATGGCAGCCGGCTTCAAGCAGATGACTTCCTCCAGGACTATACTTTATTGATCAACATCCTTCATAGTGAAGACCTAGGAAAGGACGTTGAATTTGAAGTTGTTGGTGATGCCCCGGAAAAAGTGGGGCCCAAACAAGCTGAAGATGCTGCCAAAAGCATAACCAATGGCAGTGATGATGGAGCTCAGCCCTCCACCTCCATAGGCATTTGCATCAAGTGTGGGCTTGGCATCTACGGAGCCCAGCAGGCGTGCCAGGCAATGGGGAGTCTTTATCACACTGACTGCTTCACCTGCGACTCGTGTGGGAGACGACTCCGTGGGAAGGCGTTCTACAACGTGGGTGAGAAAGTGTACTGCCAGGAGGACTTCCTGTACTCCGGGTTCCAGCAGACGGCCGACAAATGCAGCGTGTGTGGACATCTCATCATGGAAATGATCCTGCAGGCCCTGGGCAAGTCCTACCACCCAGGCTGCTTCCGGTGCTCCGTGTGCAATGAGTGCCTGGATGGGGTTCCCTTCACCGTGGACGTGGAGAACAACATCTACTGCGTGCGAGACTATCACACGGTTTTTGCACCAAAATGCGCCTCCTGTGCCCGTCCTATCCTCCCTGCACAGGGCTGCGAGACAACCATCCGTGTGGTGTCCATGGACAGAGACTACCACGTGGCATGTTACCACTGTGAGGACTGCGGGCTGCAGCTGAGCGGGGAGGAGGGACGCCGTTGCTATCCCCTGGTCAGCGTCAGGGGAGCTCCCTCCAATCAGTTTCCCACCGAGCTGCTGTCTGCAGGGGCCGGACCCCCGCGTGGAAGCTTCTATTTATTCACCGTCTGTGCCTG
